# The Political Economy of Priority Setting and Resource Allocation in European Oral Health Policy

**DOI:** 10.1177/23800844241302052

**Published:** 2024-12-19

**Authors:** Z. Sarroukh, P. Jeurissen, S. Akter, S. Listl

**Affiliations:** 1 Department of Dentistry, Quality and Safety of Oral Health Care, Radboud University Medical Center, Nijmegen, Gelderland, The Netherlands; 2 IQ Health, Radboud University Medical Center, Nijmegen, Gelderland, The Netherlands; 3 Heidelberg Institute of Global Health, Section for Oral Health, Heidelberg University Hospital, Heidelberg, Baden-Württemberg, Germany

**Keywords:** government regulation, health care rationing, health care reform, health care evaluation mechanisms, health priorities, policy making

## Abstract

**Aim:**

Pressing oral health care challenges pose prioritization dilemmas for governments. This study aimed to identify key determinants of prioritization in oral health policy in Denmark, Germany, the Netherlands, and the United Kingdom, as part of a series of the DELIVER project.

**Methods:**

A literature review based on a search of PubMed and Google Scholar articles related to these countries from January 1, 2000, to October 17, 2023, and key informant interviews with policy makers were conducted to identify key trends in oral health policy choices and determinants of priority setting and resource allocation processes.

**Results:**

A total of 249 articles were included, and 6 key informants were interviewed. The overarching focus identified was the accessibility of dental care, primarily characterized by incremental and localized programs for vulnerable groups. Supply-side arrangements consisted of adaptations to population needs, including financial incentives for providers and adjusted service delivery models such as task shifting. Several interventions of quality management were found, particularly in Germany. A funnel was produced to illustrate 3 stages driving oral health policy choices. These were political accountability to address population demand, stakeholder influence through negotiations and lobbying, and bureaucratic justification of policy innovations. While findings highlighted political attention on oral health care through public outcry, complex negotiations and limited data formed bottlenecks of prioritization.

**Conclusion:**

Prioritization in oral health policy seems to be dominated by fragmented investments in incremental services of delivery rather than synergized reforms such as granular package designs. While some contexts showed political traction for oral health policy, complex negotiations strained by interests of private professionals and challenges of limited evidence result in difficulties in constraining oral health care within public spending targets. This has placed oral health policy in a state of inertia, where insufficient public resources meet the force of content exerted by the private sector.

**Knowledge Transfer Statement:**

This study can inform policy makers and researchers to understand the various stakeholder roles in maintaining the status quo of oral health policy and the processes creating the bottlenecks preventing progression in improving oral health care systems. This understanding could lead to novel approaches to oral health policy making and the appropriate data acquisition and analysis to aid oral health policy.

## Introduction

Nowadays, more than 3.5 billion people worldwide suffer from oral diseases. A large share of these diseases is preventable; the most common being dental caries, periodontal diseases, and orodental trauma (World Health Organization [WHO] 2022). Moreover, oral health care is the third most expensive form of health care in the European Union (EU) and is forecasted to double in expenditures by 2040 (Jevdjevic et al. 2020). European countries spend 5.1% of total health care expenditure on dental care, surmounting to 90 billion dollars in 2019 (Winkelmann et al. 2022). Despite this substantial economic burden, the sector has relied heavily on out-of-pocket funding. As a result, oral health care has become one of the main drivers of catastrophic health expenditures, with many patients unable to access good-quality care (WHO 2022). Especially countries with high out-of-pocket expenditures encounter greater rates of foregone dental care due to financial barriers (Winkelmann et al. 2022). Such barriers exacerbate the disease burden of oral health as patients experience reduced quality of life and indirect costs due to productivity losses (WHO 2022). Altogether, these trends in disease burden and costs of care have raised several challenges for countries.

In response to these challenges, countries have experienced pressures to raise public resources for oral health care and increase oral health coverage. Contrary to this ever-increasing demand lies a limited public budget, confronting governments with complex prioritization questions to allocate resources differently. Various frameworks have proposed domains of health care systems that serve as policy levers to improve overall system performance. Among the most widely recognized is the WHO health system strengthening framework, consisting of 6 key areas crucial to the attainment of health care goals. The framework argues that optimizing the availability of services in line with population needs, supported by a responsive workforce, reliable information, essential medical products, efficient and equitable financing, and managed by good leadership, can strengthen health systems (WHO 2007). Other frameworks include the taxonomy of Lavis (2023) consisting of governance arrangements, financial arrangements, delivery arrangements, and implementation strategies, and the framework of Feachem et al. (2017), which distinguishes between both private and public financing and provision of health care services. While the latter synthesizes domains into broader categories, the WHO health system building blocks comprehensively capture more granular functions of health systems, facilitating the consideration of a broad range of public programs. Ultimately, decision makers must prioritize options both within and across these programs to address rising pressures bounded by public resources.

The rationalist perspective on policy decision-making postulates that resources are allocated based on a careful evaluation of the problem and a complete scope of alternative solutions (Shiffman et al. 2022). Ideally, all benefits and costs of subsequent choices are weighed systematically to ensure the highest attainable societal benefits within public resources. Such optimization of social benefits is commonly referred to as *welfare maximization* and considers both costs and effects of options through cost-benefit analyses, comparing the efficiency of various solutions. However, policy contexts are characterized by limited capacities, imperfect information, and political interests, resulting in deviations from such welfare maximization (Goddard et al. 2005). Other perspectives have therefore deviated from rational decision-making, such as the incrementalist perspective. This model describes a preference for small change in current policy to avoid complex policy making. Moreover, the punctuated equilibrium perspective emphasizes prolonged periods of limited change, interrupted by occasional radical reform due to changes in the perception of current policy as well as the entrance of new actors (Shiffman et al. 2022). Despite these contrasting perspectives, Goddard et al. (2005) argued that a rationalist perspective can be applied through models of political economy to explore seemingly irrational decisions. Political economy refers to the dynamics between governments, institutions, and economic systems that determine decision-making. As argued by the authors, these interactions may result in decisions deviating from societally optimal prioritization to align with the individual rationality of decision makers. Key models that help to understand these interactions include voting models, interest group models, and bureaucratic models. Voting models suggest that governments in democratic countries will seek the opinion of the median voter in their policy decisions to ensure elections. Interest group models explain that stakeholder groups with positions of power can steer policies in their favor as governments try to negate their opposition. Finally, bureaucratic models elaborate on the competition across bureaucracies within governments for resource allocation (Goddard et al. 2005).

As oral health expenditures continue to rise, understanding current efforts within public spending of countries to address challenges becomes increasingly important. Previous literature regarding political economy of dental care has pointed toward prioritization of smaller-scale policy programs supporting public financing of dental services. Examples include oral health promotive programs for children and coverage for marginalized groups in Canada and the United States. On the other hand, governments have refrained from extensive reforms, including coverage expansions to protect broader portions of the population. Explanations are found in the privatization of dental care and subsequent difficulty to find support among stakeholders for substantial change (Picard 2009; Quiñonez 2021). Similarly, Deal (2015) maintained that the oral health care system of the National Health Service (NHS) in the United Kingdom has seen limited meaningful change, prioritizing performance measurement, due to its liberalized market construction. A recent study by Winkelmann et al. (2022) reported on financing structures and service delivery models of oral health care systems in Europe. The study identified various extents of public financing schemes for oral health care in these countries, ranging from limited to comprehensive coverage. Importantly, while comprehensive in its selection of countries, the authors limited their scope of public policy to coverage and service provision. Moreover, the study did not investigate drivers of policy choices across these countries. Few studies have addressed the underlying processes of priority setting and resource allocation driving the status quo of prioritized policies supporting public financing of dental services across Europe.

Drawing on this existing evidence and background, this research aimed to address and inform current policy discussion through an exploratory study of the status quo as well as determinants of priority setting and resource allocation of policies that support public financing of dental services in European countries. This study adopted a rationalist perspective on policy decision-making. Based on insights from political economy, it helped to understand why, when presented with well-formulated cost-benefit analyses of the value of implementing a certain public financing model for dental care, will policy makers not adopt it? More specifically, what policy options have been prioritized instead, and can models of political economy explain barriers to adoption of fundamental change? The study focused on Denmark, Germany, the United Kingdom, and the Netherlands as these countries provide good exemplary insights into various European oral health policy systems, ranging from limited oral health care coverage to partial and comprehensive coverage systems (Winkelmann et al. 2022). The study is part of a series of the DELIVER project.

## Method

### Study Design

This study formed part of a multilevel situational analysis of the status quo of quality improvement in the DELIVER project. Further details about the EU DELIVER project are described elsewhere (Listl et al. 2024). The study focused on national oral health policy in Denmark, Germany, the Netherlands, and the United Kingdom. A literature review was used to collect insights on current policy efforts and generate an overview of policies supporting public financing of dental care in these 4 countries, in combination with key informant interviews to identify determinants of prioritized policies. Therefore, the interviews were used to understand the determinants of the status quo identified through the literature review. A thematic analysis approach was taken throughout this study as it provides flexibility and versatility that facilitate the analysis of our different types of data while integrating the theoretical underpinnings of political economy (Braun and Clarke 2006).

### Data Collection

The first part of this study aimed to identify the status quo of oral health policies prioritized in European countries. As mentioned, several frameworks exist that describe policy options for health system improvement. The WHO health system strengthening framework was used to conduct a literature review as this framework extends beyond the domains described by others, facilitating a comprehensive overview of alternative programs (WHO 2007). The service delivery building block focuses on ensuring that the right care reaches patients in need, through policies such as task shifting. The health workforce domain focused on the providers and their ability to attend to population needs, including policies addressing skill mix and training programs. The information of health systems covers the collection and dissemination of crucial health data in a country. Relevant policies in this domain included information and reporting systems. The domain of medical products focused on the availability of medicines and technologies to treat patients. Some examples of policies are guidelines and standards to promote evidence-based and safe practice. The financing of health care systems refers to raising funds and purchasing care for populations. Risk-pooling mechanisms and financial incentives for providers are 2 important examples of policy options in this building block. Governance serves as the final building block of this framework, encompassing the guidance of health systems to attain societal goals, through policies such as regulation and decentralization (WHO 2007). A Medline search through PubMed was used to identify studies describing oral health policy instruments concerning at least 1 of these 6 health system building blocks in Denmark, Germany, the Netherlands, and the United Kingdom from January 1, 2000, to October 17, 2023. Additional literature was hand searched through Google Scholar based on these findings. Details regarding the search strategy can be found in the appendix.

The second part of this study identified key determinants of priority setting and resource allocation of policies that supported public financing of dental services in European countries, to help us understand the barriers to the adoption of fundamental change. Interviews were conducted by one researcher (Z.S.) and used to gather insights on current policy priorities and the process driving priorities of oral health care. Given the limited pool of national policy makers directly involved in oral health policy, key informant interviews were conducted. Policy makers from offices of chief dental officers and government agencies in Denmark, Germany, the Netherlands, and the United Kingdom were invited to take part in semi-structured interviews. They were selected because these participants possessed an overview of policy processes in their respective countries. Interview guides were constructed based on insights from political economy, with a focus on voting models, interest group models, and bureaucratic models (Goddard et al. 2005). Voting models describe the inherent incentive of decision makers to take positions that increase their number of votes from the public and raise the probability of being elected. Interest group models highlight the positions of power among better-informed populations within society. These groups have the resources to organize themselves relatively easily and voice their opinion. This position provides them the opportunity to enforce policy decisions that lie in their interest. Moreover, positions of power are distributed within the bureaucratic structure of governments depending on budgets allocated to government bodies, as explained by bureaucratic models. These bodies may steer policy choices to increase the budget allocated to them (Goddard et al. 2005). These concepts have been translated into interview questions. The resulting interview guide can be found in the appendix. Interviews were conducted in English through Microsoft Teams and were audio-recorded. The recordings were transcribed verbatim and anonymized.

### Data Analysis

The data were deductively coded applying a thematic analysis approach. Codes and subsequent themes were informed by the theories of political economy and health system strengthening used for data collection (Braun and Clarke 2006). Patterns described in the literature related to health system domains were coded by 1 researcher (Z.S.) and categorized into prioritized health policy programs across countries. Transcripts of the interviews were uploaded to AtlasTI as well as MaxQDA to facilitate analysis. Patterns in these transcripts related to the model of political economy and health system domains were coded by 2 researchers (Z.S. and S.A.) and categorized into broader themes denoting prioritized policy programs and key determinants of priority setting. Importantly, policy programs identified through the literature and transcripts were combined into broader themes of oral health policy priorities, which could then be connected to the key determinants of prioritization as identified through the interviews. The study team engaged in continuous discussions throughout analysis to reflect on identified patterns and categorize, as well as refine, broader themes. Moreover, situational analysis as described by Clarke and Friese (2007) was applied to support analysis. Situational analysis is rooted in grounded theory and stems from symbolic interactionism, pragmatism, and constructivism. The method seeks to generate insights into social context by mapping relationships between various elements that constitute the situation of study. Given its strength in highlighting the dynamics of power underlying social contexts, the approach benefits the understanding of influence exerted by stakeholders, which is especially relevant for the political economy model. Moreover, the method is applicable to small interview studies (Clarke and Friese 2007). Therefore, situational analysis was integrated into our approach. Situational maps were built to explicate all relevant actors and aspects of policy priority setting in oral health care. Social worlds/arenas maps were used to explore relations between the various stakeholders. Positional maps were created to explore different interests and resulting positions taken by decision makers in policy priority setting. Altogether, these maps were used as tools to organize thought processes in support of the analysis.

To ensure validity and reliability of the results, intercoder agreement was monitored throughout the analysis. An approach, informed by Campbell et al. (2013), was adopted in which coders discussed discrepancies and agreed on a common code book after coding the first interview. This process was repeated for the second and third interviews. As a result, a synergized codebook was established after the first 3 interviews. The other interviews were all coded individually, after which the coders met and checked intercoder reliability. Importantly, the study team brought an expertise in health economics and health policy, enabling the authors to recognize various key phenomena of the priority setting and resource allocation of countries.

### Ethics Approval

This study was approved by the ethics commission of the Medical Faculty of Heidelberg University (S-089/2023). In addition, the study was exempted from a review by the Dutch Medical Ethical Clearance Commission (METC) and the ethics commission at Radboud University Medical Center, since participants are not subjected to actions that fall under the Medical Research Involving Human Subjects Act (WMO).

## Results

The search strategy generated 5,138 articles, of which 249 unique articles reported on oral health policy measures and were included in the analysis, as shown in the appendix. Moreover, 10 key informants were contacted, of whom 6 agreed to participate in interviews. Table 1 shows a summary of the participants’ characteristics. Three participants were female, and 3 were male. The participants were employed in the chief dental offices and government agencies for oral health policy from the 4 mentioned countries. All participants were involved in national oral health policy. Further details about the participants are not provided because of the risk of disclosure from the small key informant purposive sample. Interviews had a mean length of 58 min and were audio recorded and transcribed verbatim for analysis. Importantly, as indicated by Clarke and Friese (2007), relevant information can also be derived from small sample sizes. Key themes described in this section were identified to support the situational, social worlds/arenas, and positional maps found in the appendix. The intercoder reliability calculation based on half the interviews revealed a 90.6% agreement between the coders.

**Table 1.** Participant Characteristics.

Participant	Country	Sex
Key informant 1 (I1)	Denmark	Female
Key informant 2 (I2)	United Kingdom	Male
Key informant 3 (I3)	United Kingdom	Male
Key informant 4 (I4)	United Kingdom	Female
Key informant 5 (I5)	Germany	Male
Key informant 6 (I6)	Netherlands	Female

### Challenges of Access to Care and Incremental Policy Innovations

Table 2 shows the focal areas of oral health policy in these countries identified by combining the literature review and the informant interview findings to generate themes on the currently prioritized oral health policies. While the country contexts differed, the analysis revealed overlapping challenges experienced in these countries. Subsequent efforts in oral health policy converged into 2 patterns: the first across Denmark, the Netherlands, and the United Kingdom and the second in Germany, which seemed to diverge in its approach.

**Table 2.** Oral Health Coverage and Main Policy Instruments, Including Examples of Germany, the United Kingdom, Denmark, and the Netherlands.^a^

	Germany	United Kingdom	Denmark	Netherlands
Funding^b^	Outpatient curative care expenditure: $401.9 per capita Financed from public expenditure: 67.1% Financed from voluntary insurance: 7.5%	Outpatient curative care expenditure: $144.9 per capita Financed from public expenditure: 41.5% Financed from voluntary insurance: 7.0%	Outpatient curative care expenditure: $256.6 per capita Financed from public expenditure: 34.7% Financed from voluntary insurance: 11.3%	Outpatient curative care expenditure: $154.8 per capita Financed from public expenditure: 12.3% Financed from voluntary insurance: 66.7%
Demand-side incentives	Universal coverage: • Free basic dental services for all statutory insurance registered individuals	Universal coverage: • Free basic dental services for children • Service-dependent copayments for adults	Targeted coverage: • Free dental services for children • Age- and service-dependent subsidies for adults	Targeted coverage: • Free dental services for children
	Targeted programs: • School-based programs	Targeted programs: • School-based programs • Programs for vulnerable groups (e.g., homeless)	Targeted programs: • School-based programs • Programs for vulnerable groups (e.g., homeless, disabled)	Targeted programs: • School-based programs
Supply-side incentives	Financial arrangements: • Fees for visiting immobile patients	Financial arrangements: • Contract reforms and financial incentives	Financial arrangements: • Risk-based incentives for care	Financial arrangements: • Price deregulation and regulation
	Service delivery models: • Integration with elderly care	Service delivery models: • Integration with elderly care • Task delegation and dental teams • Referral management	Service delivery models: • Task delegation and dental teams • Risk-based care	Service delivery models: • Integration with elderly and disability care • Task delegation and dental teams
Governance and quality regulation	Centralization: • Increasing tasks for central governance	Decentralization: • Decentralized commissioning	Decentralization: • Municipal responsibility for oral health care	Decentralization: • Public oral health and health promotion by municipalities
	Quality management: • Clinical audits • Guidelines • Continuing education	Quality management: • Continuing education • Guidelines	Data management: • Oral health data collection	
	Data management: • Oral health data collection			

a Contents of the table represent a categorization of policy options in the oral health care sector based on a narrative review. A list of references collected during this review can be found in the appendix.

b Data for the year 2021, retrieved from OECD Health Statistics (2023).

#### Accessibility of oral health care

Informants reported several challenges in these countries, highlighting the multifaceted nature of policy problems in oral health care. Most informants expressed issues of affordability, accessibility, and equity (I1–I4 and I6). Patient populations struggled to pay for their oral health care expenditures, resulting in issues of accessibility, particularly among those in lower socioeconomic status populations. These problems were reported to be strongly related to issues of financing and service delivery models of countries. Oral health care systems are largely privatized, and public subsidization is limited. An example of this is Denmark, where oral health care is publicly provided to children, while the care for those older than 22 y is private. In the Netherlands, oral health care is provided by private dentists, and care is only publicly covered up to the age of 18 y. The United Kingdom has experienced a trend of dentists leaving the NHS system to deliver care in the private sector, resulting in dental deserts.

What we had then were practices that, as a business, it wasn’t making financial sense for them to be completely health service providers and they started then to reduce their health service work, and they increased their private work. (I3)

Another commonly reported challenge was the demographic trends resulting in new demands from the population (I3–I5). For example, reductions in the disease burden meant that provider contracts required adaptations to match population needs in the United Kingdom. Now that the aging population carries a significant share of the disease burden, service delivery models will see a shift in treatment patterns. A trend toward greater demands for cosmetic care was also noted.

#### Targeting vulnerable groups

The participants commonly emphasized accessibility and equity in describing the national policy perspective on quality of oral health care, alongside efficacy and epidemiological concerns (I1–I3, I5, and I6). A key theme within access formed the coverage for oral health care provided by health care systems. Notably, there was a clear focus on ensuring access to oral health care for children among the countries (I1–I4 and I6). While coverage of oral health care varied between the countries, all 4 provided at least coverage for children (Winkelmann et al. 2022). Hence, access among the younger population was one of the key priorities in national policy. Moreover, targeted programs were instruments to improve access for vulnerable population groups in the interviews and the literature review (I1, I2, I4, and I6). Denmark had implemented several school-based programs for children since the 1970s with the introduction of the Danish Act on children’s oral health and has provided outreach for frail elderly since 1994. More recently, funding has become available for underserved populations. In 2020, municipalities became responsible for organizing public programs providing oral health care to homeless patients and drug abusers (I1; Holm-Pedersen et al. 2005; Baelum 2011; Hede et al. 2019).

And the municipality program, the public municipality programs, they are also targeting the most vulnerable groups in the society that private practice are not so good in tackling. (I1)

Germany has organized dental screenings of infants and school-based programs since the 1990s (Sagheri et al. 2007). Similarly, programs in the Netherlands, such as “Keep Your Mouth Healthy” (Hou Je Mond Gezond) focus on raising oral health literacy among children (Winkelmann et al., 2022). Some examples from the United Kingdom include oral health promotion for children through “Childsmile” and “Designed to Smile” in Scotland and Wales, respectively, and outreach for elderly through Gwên am byth in Wales (I2; Chestnutt et al. 2017; Winkelmann et al., 2022).

#### Adapting the workforce and its financing schemes

A considerable share of the participants described changes in workforce arrangements as policy instruments, especially in the United Kingdom (I2–I4 and I6). Financial incentives embedded in contracts have seen adaptations to fit population and provider needs. An example of this is the contractual changes introduced in 2006 to reorient the intervention-led practice of dentistry in England toward a focus on individual risk and prevention (Bridgman and McGrady 2015).

It’s still voluntary at this stage because it’s contract reform, so practices being asked to work differently and the key component of that is they’re given simplified metrics to achieve. We feel that we’ve reduced the number of unique patients they have to see for the same contract value, but we expect the step up on prevention. (I2)

Similarly, Denmark implemented a risk stratification in its remuneration of dental checkups in 2015 to incentivize treatment based on the needs of patients (Gabel et al. 2020). In the Netherlands, free pricing was introduced to stimulate price competition among dentists. However, maximum tariffs were reintroduced soon after a substantial increase of prices was observed (Trescher et al. 2020). Efforts to stimulate oral health care for frail elderly in Germany were accompanied by fees covering visits to these patients in 2012 (Ziller et al. 2015).

Importantly, changes in workforce models were highlighted as key policy instruments (I1–I3, I5, and I6). Participants describe task shifting between oral health care providers, such as increases in the autonomy of dental hygienists in the Netherlands and the United Kingdom. Dental hygienists in the Dutch system have been able to provide preventive care to patients independently since 2006 (Northcott et al. 2013; den Boer 2020). The “Options for Change” strategy in the United Kingdom from 2002 has pushed for improved skill mixing to focus on prevention. Like the Netherlands, dental hygienists have been able to provide services independently since 2013 (Pitts 2003; Northcott et al. 2013).

There are more dental hygienists proportionately in the Community Dental Services, so they have staff who can do this [prevention] for less money. (I3)

As shown in Denmark, task shifting through dental hygienists provides cost-effective opportunities to reduce caries among children (Fejerskov et al. 2013). However, efforts of task shifting were not mentioned in the German context. In addressing the population needs, several informants expressed the opportunities of coordination between oral and overall health care (I1, I2, I4, and I5). Some examples of integration identified through the literature include the contracts between dental practices and nursing homes in Germany and the Netherlands (Nitschke et al. 2021; Weening-Verbree et al. 2021).

#### Quality regulation

Contrary to the other countries, Germany has a comprehensive coverage for oral health care where fewer challenges of access were expressed (I5; Winkelmann et al. 2022). Notably, this system is accompanied by a centralized responsibility for quality assurance within tasks of macro-level commissioning through the Joint Federal Committee (Ziller et al. 2015).

But all in all, I think the German system is very good financed, and so we have very good oral epidemiological data about the oral health status, the periodontitis cases are relatively high, but I think all in all, we live on a good island in this field, I think. (I5)

In contrast, devolved responsibilities for oral health care were a trend across Denmark, the Netherlands, and the United Kingdom (van der Poel 2006; Holmes et al. 2009; Skeie and Klock 2014). An example of this was the contract reforms in England in 2006, decentralizing the commissioning of oral health care to former primary care trusts (Holmes et al. 2009). Governance in these countries has consisted of localized responsiveness to challenges of access, characterized by interventions targeting vulnerable groups.

While fewer challenges of access were found in Germany, a particular focus on quality management of dental practices was also noted. Current efforts have been focused on continuing education for dentists and quality management systems of practices. For example, internal quality checks have been mandated since 2004, and differences between services are monitored based on standardized indicators introduced in 2010 (Ziller et al. 2015). Similarly, efforts of quality management in the United Kingdom and Denmark were also identified in the literature, referring to broader commissioning responsibilities rather than management at micro-level service delivery performed by agencies. Some examples include mandatory continuing education and guidelines in the United Kingdom and the collection of oral health data through municipalities in Denmark (Ross et al. 2012; Skeie and Klock 2014).

Altogether, these findings indicate an overarching focus on access to oral health care, particularly in countries with limited and partial coverage. The scale of policy instruments has varied, but efforts appear to have been dominated by incremental interventions through targeted programs for vulnerable populations to raise and meet the demand for oral health care as well as workforce interventions to adapt the supply side of oral health care. Moreover, we find several interventions of quality management, which formed a particular focus in Germany.

### Political Incentives of Oral Health Policy

Several themes were identified from the key informant interviews describing the priority setting of oral health policies, in pursuit of answering the second part of our research question. Following discussion within the research team, 3 key determinants were identified that could explain current policy choices of priority setting in Denmark, Germany, the Netherlands, and the United Kingdom.

#### Political accountability and points

One of the key determinants of priority setting in oral health care identified from the interviews was the role of noise in the system, stemming from different stakeholders. Vocal populations within the public have expressed dissatisfaction with current accessibility, especially in countries with limited to partial coverage. Moreover, informants explained that oral health care had received substantial media attention in recent years (I1–I4). While underlying motivations may have varied, dental workforce organizations and patient organizations will often share their views alongside media stories to amplify the noise of dissatisfaction among the population (I1–I4 and I6). This has emphasized the importance of fair representation in placing issues on the political agenda, as those without resources or organizations could be left without the ability to be heard to the same extent (I1–I3).

There are many patients that don’t have a patient association, or they don’t have a voice, they don’t have someone to raise their voice, so they’re completely forgotten, and nobody really noticed them, despite that perhaps they have much more problems than the patient association. (I1)

Importantly, the noise in the system can translate into policy as a result of political accountability. Participants expressed several forms of reactions from politicians to these public voices as they considered their role as (potentially) elected officials (I1–I4 and I6). An example of these reactions was the focus on accessibility embedded in the oral health care strategies of these countries: a commonly raised frustration (I1–I4). Resulting targeted programs for vulnerable populations aligned with these notions of accessibility. On the other hand, policies that fueled rather than controlled the noise in the system could form barriers to implementation. An example of this was water fluoridation, which, despite its evidence, has spurred vocal responses from population groups and subsequently varying reactions from politicians (I2–I4). While Germany has not experienced similar extents of public attention and media coverage, we identify a similar phenomenon. Contrary to other countries, a level of content was expressed concerning current accessibility in Germany, given the comprehensive coverage for oral health care. However, patients have been vocal about experiences with clinical errors by practitioners, forming a potential explanation for the focus on quality management in Germany.

Well, I can tell you that certainly in the past, we have considered approaches such as water fluoridation, which would be supported by the dental profession, would be supported internally within the Department of Health, by the civil servants and by the dental experts, but would not be supported by the public and would not be supported by the politicians. Partly because it’s not supported by the public. (I3)

A key consideration noted during the interviews is whether accountability is internalized by elected officials. While there may be reactions such as frustration from the public, this may not necessarily be targeted at the politicians (I2, I4–I6). Some of the tasks within health care systems are decentralized to local governments, such as health boards in the United Kingdom, transferring accountability to other stakeholders (I1–I6). The result is that this may deviate problems and public reactions away from politicians, which could reduce incentives to act at the national policy level.

Ultimately, the incentives of politicians reside in political points. In a highly complex environment with competing policy problems, various voices of dissatisfaction, and limited feasible options for innovation, politicians are inclined to opt for the easiest route toward political points. Informants describe the effort that policy takes as an important determinant of policy choices (I2, I4, and I6). Hence, raising oral health care and addressing accessibility, for example, through incremental changes to the workforce, has formed an easy approach to political points (I2 and I4).

It’s lots and lots of media attention, so, and political attention. It’s very easy to score political points with something like dentistry, it’s an easy, easy thing to raise, isn’t it. (I2)

#### Stakeholder influence through policy negotiation and lobbying

Participants described a dynamic policy process marked by several stages of stakeholder engagements with the government to form policies (I1–I6). While public views can direct political strategies, practical implications rely on government-initiated engagements. Stakeholders find opportunities to voice their opinion in decisions regarding the type of interventions and the means of implementation. Key informants expressed the importance of these engagements, as they facilitate transparency and allow governments to actively apply the feedback of stakeholders (I1, I2, I4, and I5). Moreover, participants commonly referred to these processes of engagement as negotiations (I1–I5). Several arguments are presented by different stakeholders, and the challenge for governments lies in weighing these to identify suitable choices (I1–I6).

Central to these negotiations were the varying positions occupied by stakeholders and subsequent ability to influence choices. Key stakeholders considered throughout all interviews can be categorized into 3 groups: patients, the dental workforce, and their representative organizations. Patients interests’ appear to be well-established in negotiations as governments consider the opinions of patients and often take these as a premise in their considerations (I1–I6). Hence, some of the participants noted that arguing through a patient perspective had been a valuable strategic position of dentists (I1 and I6). In addition to the patient’s interests, the dental workforce has primarily been concerned with its working conditions (I1–I6). Given their essential role in the provision of care, the workforce is able to mimic a monopolistic position in negotiations, particularly in contexts where oral health care can be provided privately. While the aim of these negotiations is to engage with various stakeholders, there appears to be a prominent role for the professional care providers during this phase of prioritization relative to citizens. Informants especially considered dentists and their financial interests reflected in these negotiations (I1–I5).

Negotiation is about compromise and it’s about everybody gets a piece of the cake, otherwise, nobody gets a piece of the cake and, you know, everyone’s miserable. (I2)

Again, resources of stakeholders are essential as they facilitate additional entry points for negotiations with governments. Workforce organizations such as the British Dental Association or the Danish Dental Association have substantial resources, providing opportunities for lobbying and serving as additional channels to instate their interests on the political agenda (I1, I2, and I3). Representation by such organizations and the resources of stakeholders are therefore shown to be an important factor in connecting stakeholders to the political world. Parallel to the trends of privatization and commercialization of oral health care, some of the key informants have expressed a growing presence of corporate organizations (I1 and I4). These could present additional political pressures from the private sector in coming years.

They can go to the parliament, then meet them and talk about the situation in order to have their interest go through and the Danish Dental Association, they do this very often. They are, but they are much more professional. They’re very professional, so they can, they meet often, meet with the politicians and they know exactly who to contact . . . . (I1)

#### Bureaucratic justification of policy innovations

One of the challenges expressed by key informants was the financial constraints within governments. Subsequently, policy investments in the public sector must compete to gain the approval of finance leads, which act as the gatekeepers of public spending (I1–I6). These finance leads include ministries of finance, health insurers, and finance departments within ministries of health, depending on the type of health care financing system and the scale of the investment. Key informants describe several advisory bodies that have served as agents to support ministries in addressing the policy agenda. Their engagement in policy revolved around the justification of innovations to be able to implement changes (I1–I6). Therefore, the ability to defend policies within this competition of bureaucratic justification was a core theme within priority setting identified in this research.

Something like that is a good example where when it’s well evaluated properly, you answer every criticism you can and that was on the forefront rather than on the back foot, and that approach was taken. (I2)

As expressed by the informants, this process commonly relied on the data and evidence available. Assessments of suitable policy interventions commonly considered impact on oral health and equity. Key informants suggested that there was a demand for broader considerations providing stronger arguments, such as the effect on overall health and quality of life (I1 and I5). However, these data have not been as readily available in oral health care. Limited data as well as dynamic policy environments described by the informants have demanded ad hoc approaches to evidence synthesis. Advisors have resorted to evidence from existing systems, using epidemiological data, data on health care services, or questionnaire data, which in some contexts are assessed retrospectively after the implementation of an intervention (I1–I6). As mentioned, costs of policy innovations form essential considerations for policy choices, which is why data on oral health care costs is also frequently used (I1–I3, I5, and I6). However, more advanced forms of evaluations such as cost-effectiveness analyses have not been as widely used in the oral health care sector. Commonly cited arguments for this among the informants were the limitations in resources to perform these (I1, I3, I4, and I5). Therefore, we find several challenges of evidence that complicate tradeoffs between policies.

. . . so that would give us an idea of the effectiveness and the cost effectiveness, so if that information existed that’s what, you know that would be factored in, but very often that isn't necessarily there . . . . (I3)

Viewing all themes congruently, efforts to expand political points have dictated the priority setting of oral health policy in the countries analyzed in this research. While stakeholders have been able to find opportunities to steer policy in their favor, suggested policies are met by necessary checks and balances of financial gatekeepers of governments. Ultimately, the evidence must justify the investment. This process can be depicted as a funnel, shown in the Figure. While oral health care has gained traction in relevance for politicians, a bottleneck of priority setting and resource allocation has been created by complex negotiations and evidence deficits.

**Figure.**
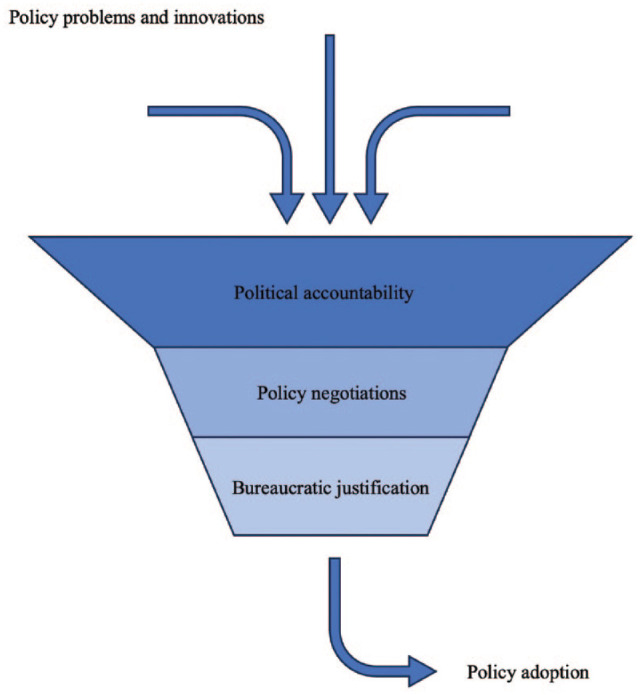
Key determinants of oral health policy choices.

## Discussion

The aim of this study was to identify determinants of the priority-setting processes that have set the stage for current oral health policy efforts in Denmark, Germany, the Netherlands, and the United Kingdom. While countries varied in health care systems and subsequent strategies, there was an overlapping focus on the accessibility of oral health care. Strategies of these countries converged in several targeted programs to reach vulnerable populations. Moreover, the findings emphasized several adaptations in models of service delivery as well as financial arrangements to address population needs. Policy efforts were also found in quality regulation. Given the limited public resources and privatized nature of dentistry, policy change has been met with resistance and primarily consisted of incremental interventions focused on targeted populations.

Several key determinants were identified underlying the priority-setting process of oral health policy. Importantly, key informant interviews highlighted the relevance of public voices that could steer political accountability as well as subsequent policy choices to manage the noise in the system. Moreover, stakeholders found ample opportunities to express their interests, denoting the negotiation process inherent to policy. While patient interests remained central in these negotiations, dentists held strong positions through their substantial resources to bargain in policy choices. These positions were especially highlighted in countries characterized by mixed private and public service provision systems, as dentists were able to find alternative sources of income in the private sector when publicly regulated systems inadequately meet their demands. Ultimately, financial implications of oral health policy investments must be justified as they compete for public funds. Given the current limited availability of data in health policy and the oral health care context specifically, policy choices have relied on pragmatic approaches to evidence synthesis, explaining a potential reluctance toward substantial investments in reforms.

These findings suggest that the models of political economy can help explain priority setting in oral health policy. In line with voting models, decision-makers appear to have prioritized oral health policies that present opportunities for political points, pursuing votes through public strategies that advocate accessibility. Subsequent selection of policy programs to address noise in the system occurs through negotiation, ultimately aligning with interests of stakeholders able to voice their opinion, as also described in interest group models. Investment in policy programs entails resource allocation to government bodies through rigorous competition between bureaucracies. Such circumstances generate incentives for decision-makers to favor policies more likely to secure investment, corresponding to bureaucratic models. These processes pose bottlenecks that permeate smaller-scale programs to be favored by decision makers, as shown in the Figure. Altogether, the model of political economy helps us understand the limited traction for substantive reform.

The lack of systematic change in oral health policy could indicate a balance between, on one hand, limitations in public financial resources and evidence to favor substantial investment and, on the other hand, the resistance posed by a preference for the status quo among the private sector. The result is that governments rely on smaller-scale programs and targeted interventions to address unmet needs. Challenges emphasized in previous literature on the political economy of oral health policy align with our findings. Deal (2015) attributed the limited fundamental change in NHS dentistry to notions of neoliberalism that have seeped into dental research agendas, commissioning, and the practice of dentistry. The government has limited its role in oral health policy to the measurements of performance and introduced market principles in pursuit of cost containment. The inadequate design of these market mechanisms has left the sector in a state that lacks the necessary construction to address fundamental needs. While signs of resistance from the profession to substantial change are acknowledged, Deal explains that the practice of dentistry has also fallen victim to this construction of the market. Quality of dental care has been put at stake as dentists experience pressures of standardization exerted by governmental focus on monitoring performance while simultaneously attending to the demand of empowered customers in the market, unable to address true clinical needs. Ultimately, dissatisfaction is found at every level of the system, each of which blames the other for the status quo (Deal 2015). Compromise between the stakeholders appears to lie at the center of policy change, as shown by Franzon et al. (2017). Their study explored politics surrounding dental care reforms in Sweden, where a large share of dental care is provided by the private sector. The authors identify 4 major reforms in the Swedish oral health care system between 1974 and 2016. Substantial change during reform was facilitated by consensus on interventions that met the interests of key stakeholders (Franzon et al. 2017). Hence, ensuring satisfaction among interest groups through negotiation forms a key determinant of prioritization, as came forward in our results.

Similar challenges of liberalized dental care markets echo across the pond. Quiñonez (2021) described a safety net construction of oral health policy in Canada. The dental profession has been resistant to the inclusion of dental care into public schemes since its conception, emphasizing the importance of the individual responsibility of patients. In line with their economic and political interests, the profession has argued that governments should limit their role to the protection of children and marginalized groups instead. Whereas most of the government’s dental expenditures were invested in public health programs in 1980, by 2005 the government shifted its focus to social assistance programs delivered by private practitioners. The profession’s influence is also apparent in service-level prioritization, as dentists have adapted the amount of publicly insured patients they treat at their practice based on the extent to which the public insurance aligns with the preferences of the profession (Quiñonez 2021). This narrative appears to be recurrent in the literature. In a study about rationing of dental services in Ontario, McKay (2013) found a preference for individual responsibility among dentists stemming from the economic interests instilled in privatized markets. Furthermore, the United States has seen comparable developments of oral health policy. Picard (2009) explained that dentists emphasized notions of individual responsibility to ensure that the profession would maintain its entrepreneurial independence in the privatized dental care market. Government involvement was argued to harm the independence of citizens. While public school–based programs for children were initially endorsed by the profession, this changed as threats to their economic interests and autonomy were perceived. As potential coverage for dental care became imminent in the 1960s, dentists once again voiced their dissatisfaction with the potential impediment of American values, posing barriers to policy change (Picard 2009). Altogether, in line with our findings the literature shows that privatization has resulted in complex dynamics between stakeholders, providing limited room for oral health policy beyond the implementation of safety nets.

Interestingly, the findings appeared to be consistent across liberalized markets for medical care. The United States has an overall health care system characterized by its privatized market structure. Financing and provision of health care services are dominated by the private sector. As a result, the role of the government has been limited to ensuring a safety net to vulnerable groups whose needs cannot be met by the market, including smaller-scale programs such as Medicare and Medicaid. However, challenges of access persist as 27.5 million citizens remain uncovered (Tikkanen et al. 2020). While public targeted programs capture some of the deadweight loss of privatized health care markets, gaps in accessibility extend beyond the outreach of these programs.

This study had several limitations. Importantly, it used the available scientific literature as proxies for current oral health policies. Therefore, policies that have received no attention from the scientific literature could be missing in the results. However, the aim of this exercise in this study was to explicate important trends in oral health policy investment choices, adding to the already available literature on oral health care financing schemes of European countries (Winkelmann et al. 2022). Another limitation was the small number of key informants directly involved in oral health policy that could be interviewed for this study. However, as mentioned by Clarke and Friese (2007), small sample sizes can provide relevant insights, especially given that the sample consisted of key informants with a unique overview of the oral health policy landscape. Importantly, saturation was noted during the analysis as arguments provided by the key informants were recurrent, and no particularly new insights were derived from the last interview. Moreover, while contexts vary, the literature review revealed convergence in oral health policy strategies across the countries. This indicated potential homogeneity in European oral health policy that could strengthen the relevance of our findings despite the relatively small sample (Hennink and Raiser 2022). Finally, potential biases in the key themes regarding oral health care and policy goals may have resulted from the inherent predispositions of participants given their role as policy makers in this sector.

While oral health care is gaining traction on the global political agenda (WHO 2022), negotiations with stakeholders and the evidence synthesis for investment choices pose bottlenecks to processes of policy choices and priority setting. As a result, oral health care has seen limited systematic reforms to address upcoming challenges, proving limited oral health system resilience. This study has highlighted underlying processes of these bottlenecks of priority setting in oral health care. The policy implications of these results reside in uptake of dentistry within publicly financed schemes, allowing for regulation of oral health care that could address the inertia posed by the workforce. Such measures could negate the potentially disproportionate position of power we find among the workforce of these countries, facilitating greater change in oral health policy. Moreover, future studies could focus on processes of policy choices that could relieve these bottlenecks.

## Author Contributions

Z. Sarroukh, contributed to conception, design, data acquisition, analysis, and interpretation, drafted and critically revised the manuscript; P. Jeurissen, contributed to conception, design, data analysis and interpretation, critically revised the manuscript; S. Akter, contributed to data analysis and interpretation, critically revised the manuscript; S. Listl, contributed to conception, design, data acquisition, analysis and interpretation, critically revised the manuscript. All authors gave final approval and agree to be accountable for all aspects of the work.

## Supplemental Material

sj-docx-1-jct-10.1177_23800844241302052 – Supplemental material for The Political Economy of Priority Setting and Resource Allocation in European Oral Health Policy Supplemental material, sj-docx-1-jct-10.1177_23800844241302052 for The Political Economy of Priority Setting and Resource Allocation in European Oral Health Policy by Z. Sarroukh, P. Jeurissen, S. Akter and S. Listl in JDR Clinical & Translational Research
