# Supplementary material for: The Political Economy of Priority Setting and Resource Allocation in European Oral Health Policy
Source: JDR Clin Trans Res. 2024 Dec 19;10(4):372–84. doi: 10.1177/23800844241302052 (PMC12402516; doi:10.1177/23800844241302052)
Supplement: sj-docx-1-jct-10.1177_23800844241302052 – Supplemental material for The Political Economy of Priority Setting and Resource Allocation in European Oral Health Policy [file sj-docx-1-jct-10.1177_23800844241302052.docx]

**Appendix for article titled: The political economy of priority setting and resource allocation in European oral health policy**

Authors: Ziade Sarroukh, Patrick Jeurissen, Shaila Akter, Stefan Listl

**Search strategy and results**

Key words and Boolean operators in our search strategy included the following: ((oral health care) OR (dental health care)) AND ((health system) OR (service delivery) OR workforce OR (health information) OR (medical products) OR financing OR governance) AND (denmark OR germany OR netherlands OR (united kingdom)). Articles published between 01-01-2000 and 17-10-2023 were included if they described policy instruments or investment options in respective countries.

Appendix Figure 1: Flow diagram of literature review

Excluded articles that did not address policy in relevant countries

(n=654)

Records excluded

(n=4,227)

Articles included in study

(n=249)

Full text articles assessed for eligibility

(n=903)

Record titles and abstracts screened

(n=5,130)

Records after duplicated removed

(n=5,130)

Additional records identified through hand searching

(n=4)

Records identified through database searching

(n=5,134)

**Interview guide**

1. **Introduction**
   1. “Hello Mr. / Mrs. welcome to our interview, thank you for being here”
   2. “My name is _ and I am a researcher at the Radboudumc, I will be conducting today’s interview and you can contact me or my colleagues if any questions arise”
   3. “This study investigates current efforts of quality improvement in oral healthcare at the national policy level”
   4. “I will ask you some questions concerning quality improvement efforts. This is meant to be an open discussion, so please answer according to your own perceptions. Please don’t hesitate to say anything that comes to mind even if you think it’s not relevant to us.
   5. Information: what data will be gathered, how it will be processed, main points of the information leaflet must be covered (e.g. voluntary participation, possibility of withdrawal of consent at any time, no repercussions if study is left before the end, data protection, rights)
   6. “I will start recording now, I will let you know when the recording is over.”
   7. “Could you please introduce yourself: What is your name, your institutions / organization / etc., your role within this, …?” (this section should be very short)
2. **Open questions in the beginning of the interview**

Status quo of policy activity and public’s opinion

- What is the government’s publicly endorsed definition of quality of oral health care?
- What have been the main problems in the oral health care sector over the past two decades?
- What has been the public’s reaction to these problems?
  - To what extent have these reactions reached the media?
  - To what extent did these reactions gain the interests of political parties?
- Could you describe recent policy interventions that have marked the current organization of the oral health system?
- What problems have these policy interventions addressed?
  - What problems remain?
  - Why these problems?

Stakeholder interests

- What stakeholders have been involved in oral health policy decisions?
- How have the stakeholders differed in their view on the oral health care problems and policies?
- What stakeholder interests do these views reflect?
- How is political influence distributed among the stakeholder groups?

Policy decision-making and evidence-base

- Who makes the recommendations for oral health policies? (advisory agency or government body)
- What methodology is used to evaluate oral health policies in your country?
- What evidence is collected for the evaluation of oral health policies?
- Which stakeholder views may be sought for the decision-making process?
- What other criteria may be considered (by policymakers) during evaluation and tradeoffs of policies?
- Where has the decision-making power lied in oral health policies?
  - How is influence distributed within the bureaucratic structure of the government?
  - What role has this distribution of influence played in oral health policy decision-making?

1. **Inquiries**
   1. Fact checking
   2. Interpretative checking
      1. “Did I understand it correctly that _?”
   3. Before not mentioned information
2. **Closing**
   1. Summarize some general themes and insight that came out of the conversation and give participants an opportunity for any final words
   2. Thank them for their input
   3. Inform participant when recording is over
   4. Inform them how the data will be used and explain how the results will be disseminated

**Situational analysis maps**

Appendix Figure 2: Situational map and relational mapping


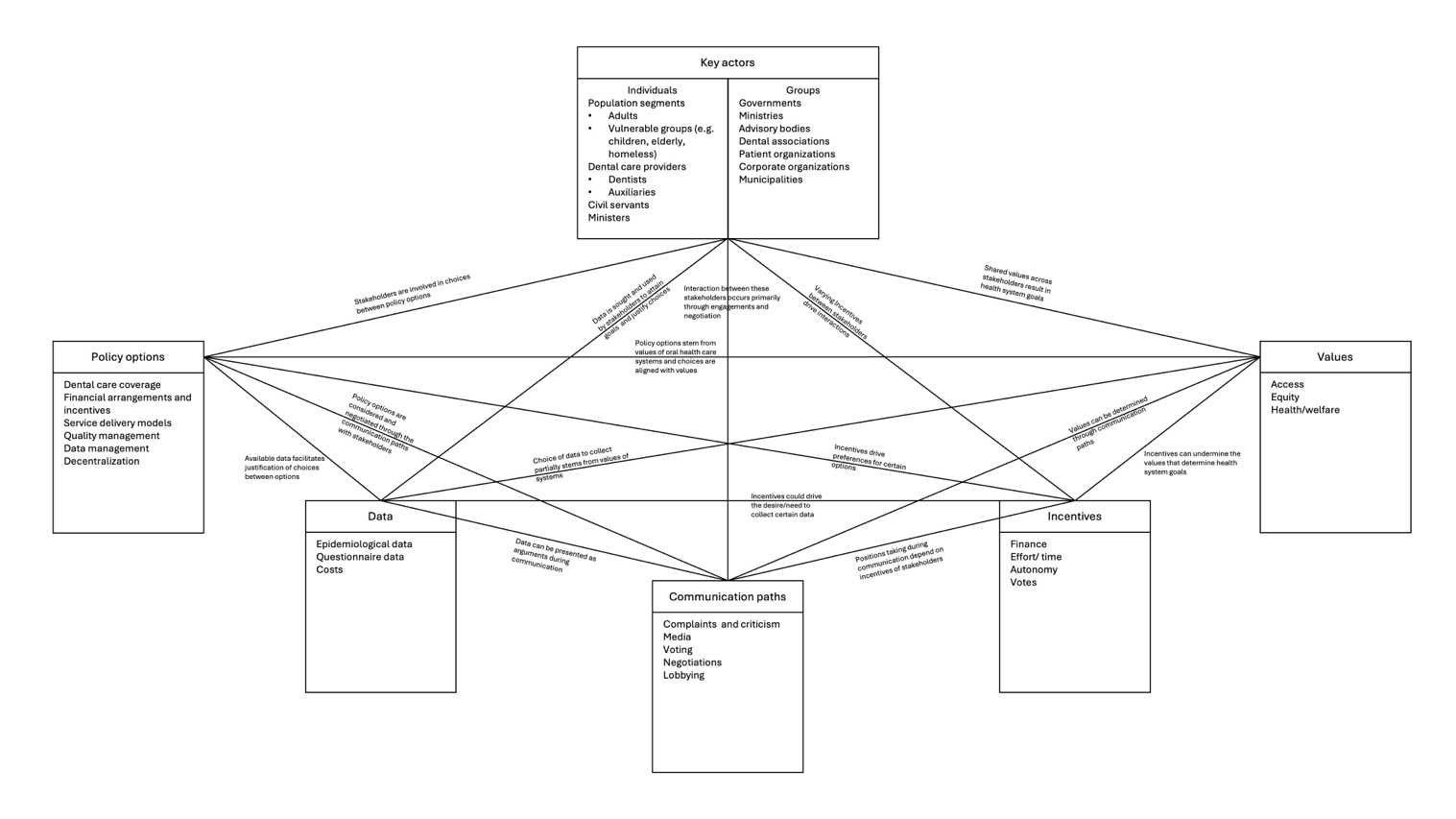


Appendix Figure 3: Social worlds/arenas map


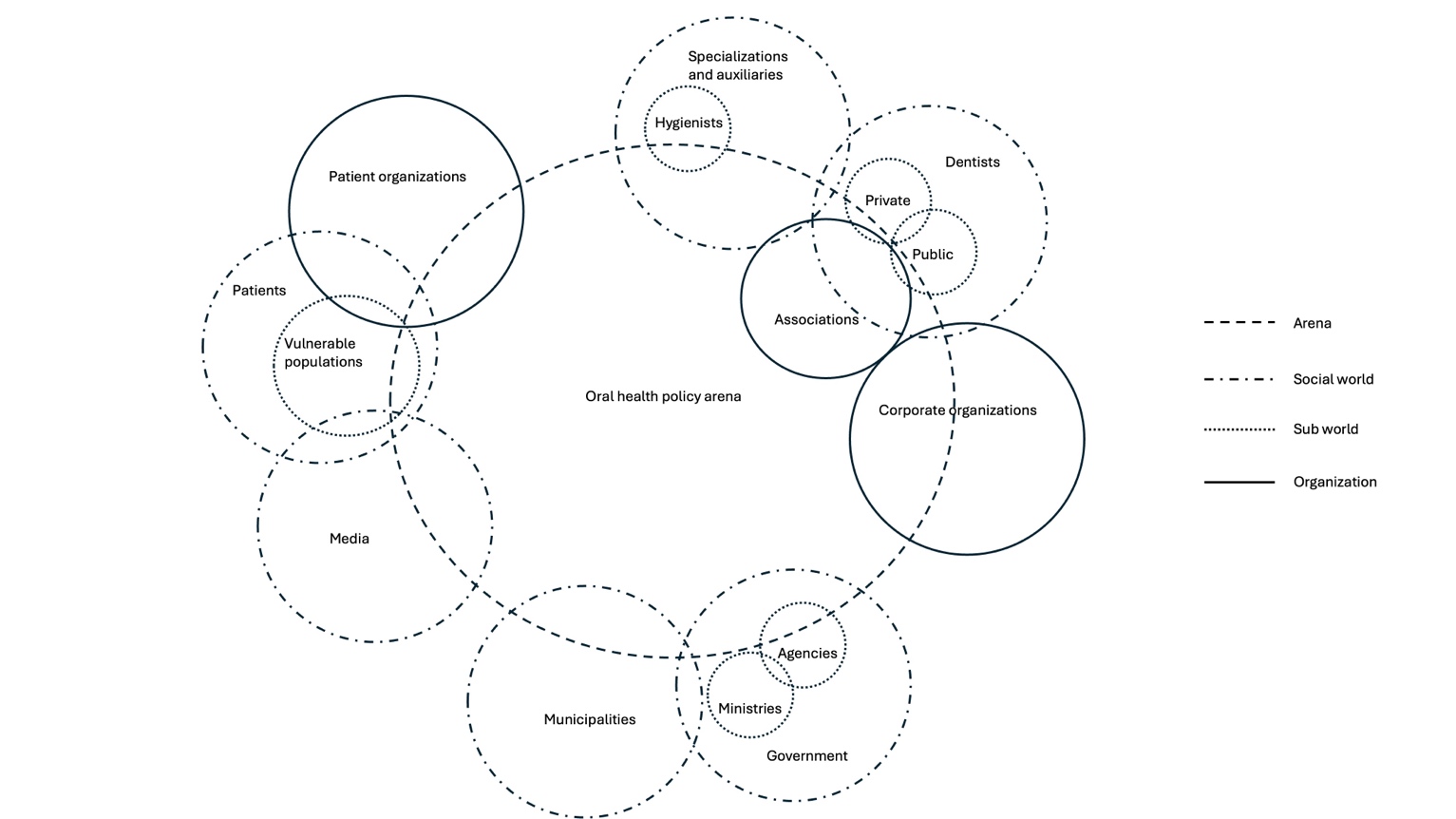


Appendix Figure 4: Positional map


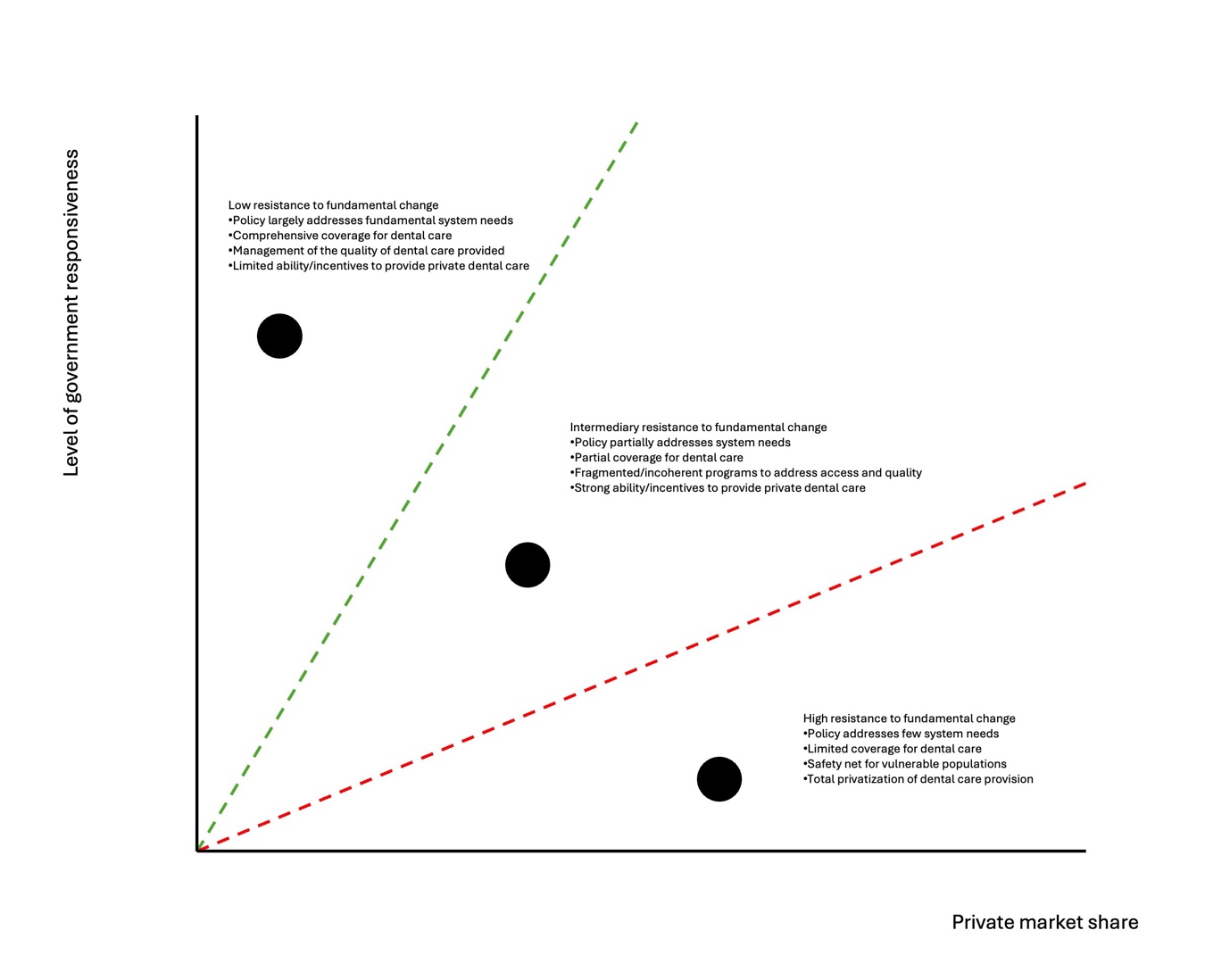


**References narrative review**

Dentistry funding in Wales to be ring-fenced until 2012, minister announces. 2008. Br Dent J. 205(3):116.

Investment of 75m pounds sterling for dentistry in Scotland. 2008. Br Dent J. 205(4):170.

New contract failing to improve dental services say MPs, as health committee urges review of UDAs. 2008. Br Dent J. 205(1):7.

Private dentistry to be regulated in Wales. 2008. Br Dent J. 205(11):586.

Fees drop as BDA encourages consistency in commissioning. 2011. Br Dent J. 210(8):347.

Online initiative to improve oral health of the elderly. 2015. Br Dent J. 218(6):323.

A NC, Robertson S, Dyer TA, Balmer RC, Fayle SA. 2010. An evaluation of paediatric dental general anaesthesia in Yorkshire and the Humber. Br Dent J. 209(12):E20.

Ahmad B, Landes D, Moffatt S. 2018. Dental public health in action: Barriers to oral healthcare provision for older people in residential and nursing care homes: A mixed method evaluation and strategy development in county Durham, North East England. Community Dent Health. 35(3):136-139.

Al Dehailan L, Martinez-Mier EA. 2019. Prevention program including fluoride varnish and 1450-ppm fluoride toothpaste targeting young children in clinical setting in UK did not stop sental caries from developing but slowed lesion progression. J Evid Based Dent Pract. 19(2):207-209.

Alani A, Bishop K. 2012. Contemporary issues in the provision of restorative dentistry. Br Dent J. 213(4):163-170.

Albrecht M, Isenbeck F, Kasper J, Mühlhauser I, Steckelberg A. 2016. The foundation in evidence of medical and dental telephone consultations. Dtsch Arztebl Int. 113(22-23):389-395.

Al-Haboubi M, Newton P, Gallagher JE. 2016. Meeting patient and professional needs: Views of stakeholders on a training initiative for DwSIs in endodontics in London. Prim Dent J. 5(2):54-65.

Ali K, Tredwin C, Kay E, Slade A. 2016. Stakeholders' perceptions about a newly established dental school with a problem-based, student-led, patient-centered curriculum: A qualitative study. J Dent Educ. 80(3):291-300.

Allin S, Farmer J, Quiñonez C, Peckham A, Marchildon G, Panteli D, Henschke C, Fattore G, Lamloum D, Holden ACL, Rice T. 2020. Do health systems cover the mouth? Comparing dental care coverage for older adults in eight jurisdictions. Health Policy. 124(9):998-1007.

Andersen LB, Bech M, Lauridsen J. 2012. Political or dental power in private and public service provision: A study of municipal expenditures for child dental care. Health Econ Policy Law. 7(3):327-342.

Anderson JA, Brewer A, Creagh D, Hook S, Mainwaring J, McKernan A, Yee TT, Yeung CA. 2013. Guidance on the dental management of patients with haemophilia and congenital bleeding disorders. Br Dent J. 215(10):497-504.

Anjrini AA, Kruger E, Tennant M. 2014. International benchmarking of hospitalisations for impacted teeth: A 10-year retrospective study from the United Kingdom, France and Australia. Br Dent J. 216(7):E16.

Anopa Y, McMahon AD, Conway DI, Ball GE, McIntosh E, Macpherson LM. 2015. Improving child oral health: Cost analysis of a national nursery toothbrushing programme. PLoS One. 10(8):e0136211.

Baâdoudi F, Maskrey N, Listl S, van der Heijden GJ, Duijster D. 2016. Improving oral healthcare: Towards measurement? Br Dent J. 221(9):547-548.

Baelum V. 2011. Dentistry and population approaches for preventing dental diseases. J Dent. 39 Suppl 2:S9-19.

Bahrami M, Deery C, Clarkson JE, Pitts NB, Johnston M, Ricketts I, MacLennan G, Nugent ZJ, Tilley C, Bonetti D, Ramsay C. 2004. Effectiveness of strategies to disseminate and implement clinical guidelines for the management of impacted and unerupted third molars in primary dental care, a cluster randomised controlled trial. Br Dent J. 197(11):691-696; discussion 688.

Baird WO, Jackson RJ, Worthington LS, Robinson PG. 2007. Perspectives of dentists in primary care ahead of the 'new ways of working'. Br Dent J. 202(10):E24; discussion 614-615.

Balasooriyan A, Dedding C, Bonifácio CC, Van Der Veen MH. 2022. Professionals’ perspectives on how to address persistent oral health inequality among young children: an exploratory multi-stakeholder analysis in a disadvantaged neighbourhood of Amsterdam, the Netherlands. BMC Oral Health. 22(1).

Ball GE. 2008. Out-of-hours emergency dental services in Scotland--a national model. Br Dent J. 205(9):485-487.

Bedi R. 2006. Challenges to dental access - England as a case study. Community Dent Oral Epidemiol. 34(3):222-224.

Belsi A, Gonzalez-Maffe J, Jones K, Wright D, Gallagher JE. 2013. Care home managers' views of dental services for older people living in nursing and residential homes in inner city London. Community Dent Health. 30(2):77-82.

Best H, Newton T. 2005. Evaluation of the personal dental services (wave 1) for Lambeth, Southwark and Lewisham primary care trusts--part 1: Retrospective analyses of registration data and access issues. J Eval Clin Pract. 11(3):219-227.

Best H, Newton T. 2005. Evaluation of the personal dental services (wave 1) for Lambeth, Southwark and Lewisham primary care trusts--part 2: Retrospective analyses of treatment and other dental record data. J Eval Clin Pract. 11(3):229-236.

Bhatti A, Gray-Burrows KA, Giles E, Rutter L, Purdy J, Zoltie T, West RM, Pavitt S, Marshman Z, Day PF. 2021. "Strong teeth": The acceptability of an early-phase feasibility trial of an oral health intervention delivered by dental teams to parents of young children. BMC Oral Health. 21(1):138.

Bhatti A, Wray F, Eskytė I, Gray-Burrows KA, Owen J, Giles E, Zoltie T, Smith V, Pavitt S, West R et al. 2022. Habit (health visitors delivering advice in Britain on infant toothbrushing): A qualitative exploration of the acceptability of a complex oral health intervention. BMC Prim Care. 23(1):55.

Birch S, Anderson R. 2005. Financing and delivering oral health care: What can we learn from other countries? J Can Dent Assoc. 71(4):243, 243a-243d.

Bonetti DL. 2014. Evidence not practised: The underutilisation of preventive fissure sealants. Br Dent J. 216(7):409-413.

Bonetti DL, Clarkson JE, Elouafkaoui P, Stirling DA, Young L, Templeton AR. 2014. Managing patients on bisphosphonates: The practice of primary care dentists before and after the publication of national guidance. Br Dent J. 217(12):E25.

Borrie FR, Elouafkaoui P, Bearn DR. 2013. A Scottish cost analysis of interceptive orthodontics for thumb sucking habits. J Orthod. 40(2):145-154.

Boyle S. 2011. United kingdom (England): Health system review. Health Syst Transit. 13(1):1-483, xix-xx.

Bradley M, Black P, Noble S, Thompson R, Lamey PJ. 2010. Application of teledentistry in oral medicine in a community dental service, n. Ireland. Br Dent J. 209(8):399-404.

Bridgman C, McGrady MG. 2015. Clinical leadership and prevention in practice: Is a needs led preventive approach to the delivery of care to improve quality, outcomes and value in primary dental care practice a realistic concept? BMC Oral Health. 15 Suppl 1(Suppl 1):S2.

Brocklehurst P, Birch S, McDonald R, Tickle M. 2013. Determining the optimal model for role-substitution in NHS dental services in the United Kingdom. BMC Oral Health. 13:46.

Brocklehurst P, Macey R. 2015. Skill-mix in preventive dental practice--will it help address need in the future? BMC Oral Health. 15 Suppl 1(Suppl 1):S10.

Buckingham S, John JH. 2017. Outcomes and costs of pre-school and school-based fluoride varnish pilots. Br Dent J. 222(8):591-594.

Burnham R, Bhandari R, Bridle C. 2011. Changes in admission rates for spreading odontogenic infection resulting from changes in government policy about the dental schedule and remunerations. Br J Oral Maxillofac Surg. 49(1):26-28.

Calcoen P, van de Ven W. 2018. How can dental insurance be optimized? Eur J Health Econ. 19(4):483-487.

Cannell P. 2005. Linking clinical audit in general dental services to primary care trust clinical governance -- progress report of an approach used in Southend. Prim Dent Care. 12(1):23-26.

Cannell PJ. 2009. Evaluation of the end user (dentist) experience of undertaking clinical audit in a pct-led NHS modernisation agency pilot scheme. Prim Dent Care. 16(4):168-176.

Cassie H, Treweek S, McKee L, Ramsay C, Young L, Clarkson J. 2022. 'Well, in dentistry the dentist is always the boss': A multi-method exploration of which organisational characteristics of dental practices most influence the implementation of evidence-based guidance. BMJ Open. 12(8):e059564.

Chestnutt IG, Chadwick BL, Hutchings S, Playle R, Pickles T, Lisles C, Kirkby N, Morgan MZ, Hunter L, Hodell C et al. 2012. Protocol for "seal or varnish?" (sov) trial: A randomised controlled trial to measure the relative cost and effectiveness of pit and fissure sealants and fluoride varnish in preventing dental decay. BMC Oral Health. 12:51.

Chestnutt IG, Davies L, Thomas DR. 2009. Practitioners' perspectives and experiences of the new National Health Service dental contract. Br Dent J. 206(9):E18; discussion 476-477.

Chestnutt IG, Playle R, Hutchings S, Morgan-Trimmer S, Fitzsimmons D, Aawar N, Angel L, Derrick S, Drew C, Hoddell C et al. 2017. Fissure seal or fluoride varnish? A randomized trial of relative effectiveness. J Dent Res. 96(7):754-761.

Chestnutt IG, Thomas DR, Patel R, Treasure ET. 2007. Perceptions and attitudes to a fundamental reform of general dental services in Wales. Prim Dent Care. 14(1):13-18.

Clarkson JE, Pitts NB, Bonetti D, Boyers D, Braid H, Elford R, Fee PA, Floate R, Goulão B, Humphris G et al. 2018. Interval (investigation of nice technologies for enabling risk-variable-adjusted-length) dental recalls trial: A multicentre randomised controlled trial investigating the best dental recall interval for optimum, cost-effective maintenance of oral health in dentate adults attending dental primary care. BMC Oral Health. 18(1):135.

Clarkson JE, Pitts NB, Fee PA, Goulao B, Boyers D, Ramsay CR, Floate R, Braid HJ, Ord FS, Worthington HV et al. 2021. Examining the effectiveness of different dental recall strategies on maintenance of optimum oral health: The interval dental recalls randomised controlled trial. Br Dent J. 230(4):236-243.

Clarkson JE, Pitts NB, Goulao B, Boyers D, Ramsay CR, Floate R, Braid HJ, Fee PA, Ord FS, Worthington HV et al. 2020. Risk-based, 6-monthly and 24-monthly dental check-ups for adults: The interval three-arm rct. Health Technol Assess. 24(60):1-138.

Clarkson JE, Turner S, Grimshaw JM, Ramsay CR, Johnston M, Scott A, Bonetti D, Tilley CJ, Maclennan G, Ibbetson R et al. 2008. Changing clinicians' behavior: A randomized controlled trial of fees and education. J Dent Res. 87(7):640-644.

Coles E, Freeman R. 2016. Exploring the oral health experiences of homeless people: A deconstruction-reconstruction formulation. Community Dent Oral Epidemiol. 44(1):53-63.

Cope AL, Bannister C, Karki A, Harper P, Allen M, Jones R, Peddle S, Walters B, Chestnutt IG. 2022. The development and application of a chairside oral health risk and need stratification tool in general dental services. J Dent. 123:104206.

Cope AL, Roper R, Chestnutt IG, Karki AJ. 2019. Exploring the feasibility of using routinely collected data to produce antibiotic prescribing profiles for general dental practitioners in Wales. Community Dent Health. 36(3):177-180.

Csikar JI, Douglas GV, Pavitt S, Hulme C. 2016. The cost-effectiveness of smoking cessation services provided by general dental practice, general medical practice, pharmacy and NHS stop smoking services in the north of England. Community Dent Oral Epidemiol. 44(2):119-127.

Cure RJ. 2019. Experiences of the challenges of undertaking an orthodontic needs assessment within the national health service in England. J Orthod. 46(2):143-147.

Currie CC, Stone SJ, Brocklehurst P, Slade G, Durham J, Pearce MS. 2022. Dental attendances to general medical practitioners in Wales: A 44 year-analysis. J Dent Res. 101(4):407-413.

Currie RB, Pretty IA, Tickle M, Maupomé G. 2012. Conundrums in health care reform: Current experiences across the North Atlantic. J Public Health Dent. 72(2):143-148.

Curtis S, Gurveer J, Gallagher JE. 2019. Clinical dental technicians in the United Kingdom: A qualitative understanding of the experiences, of practices and challenges of communication for dental professionals. Gerodontology. 36(4):405-416.

Daly B, Newton JT, Batchelor P. 2010. Patterns of dental service use among homeless people using a targeted service. J Public Health Dent. 70(1):45-51.

Daly B, Newton T, Batchelor P, Jones K. 2010. Oral health care needs and oral health-related quality of life (ohip-14) in homeless people. Community Dent Oral Epidemiol. 38(2):136-144.

Davies BJ, Macfarlane F. 2010. Clinical decision making by dentists working in the NHS general dental services since April 2006. Br Dent J. 209(10):E17.

Davies-Slowik J, Firmstone V, Frame J. 2011. Educational support for the dental workforce: A review of the first five years of retaining and returning advisers in England. Br Dent J. 210(5):E6.

de Oliveira CM. 2003. The planning, contracting and monitoring of orthodontic services, and the use of the IOTN index: A survey of consultants in dental public health in the United Kingdom. Br Dent J. 195(12):704-706; discussion 696.

den Boer JCL, van der Sanden WJM, Bruers JJM. 2020. Developments in oral health care in the Netherlands between 1995 and 2018. BMC Oral Health. 20(1):192.

Douglas GV, Ramsdale MP, Vinall-Collier K, Csikar JI. 2016. Using high fluoride concentration products in public policy: A rapid review of current guidelines for high fluoride concentration products. Caries Res. 50 Suppl 1:50-60.

Dowey P. 2017. Improving care for bariatric dental patients in north Wales. Community Dent Health. 34(2):80-83.

Dragheim E, Petersen PE, Kalo I, Saag M. 2000. Dental caries in schoolchildren of an Estonian and a Danish municipality. Int J Paediatr Dent. 10(4):271-277.

Dyer TA, Humphris G, Robinson PG. 2010. Public awareness and social acceptability of dental therapists. Br Dent J. 208(1):E2; discussion 16-17.

Eaton KA. 2005. Dentists with special interests (DwSIs): Further developments. Prim Dent Care. 12(3):75-77.

Eaton KA. 2012. 2012: A time for change. Prim Dent Care. 19(1):3-4.

Eaves J, Gnich W. 2013. Can programme theory be used as a 'translational tool' to optimise health service delivery in a national early years' initiative in Scotland: A case study. BMC Health Serv Res. 13:425.

Edwards DM, Merry AJ, Pealing R. 2002. Disability part 3: Improving access to dental practices in merseyside. Br Dent J. 193(6):317-319.

Edwards K, Rae J, Rolland S, Vernazza CR. 2022. The value of adult orthodontics: Do the public's willingness-to-pay values reflect the profession's? J Orthod. 49(2):113-121.

Ekstrand KR, Christiansen ME, Qvist V, Ismail A. 2010. Factors associated with inter-municipality differences in dental caries experience among Danish adolescents. An ecological study. Community Dent Oral Epidemiol. 38(1):29-42.

Eskytė I, Gray-Burrows KA, Owen J, Sykes-Muskett B, Pavitt SH, West R, Marshman Z, Day PF. 2021. Organizational barriers to oral health conversations between health visitors and parents of children aged 9-12 months old. Front Public Health. 9:578168.

Fejerskov O, Escobar G, Jøssing M, Baelum V. 2013. A functional natural dentition for all--and for life? The oral healthcare system needs revision. J Oral Rehabil. 40(9):707-722.

Firmstone VR, Bullock AD, Jackson D, Manning R, Davies-Slowik J, Frame JW. 2010. Using evaluation to enhance educational support for dental teams in the UK. J Dent Educ. 74(8):892-901.

Gabel F, Kalmus O, Rosing K, Trescher AL, Listl S. 2020. Implementation of altered provider incentives for a more individual-risk-based assignment of dental recall intervals: Evidence from a health systems reform in Denmark. Health Econ. 29(4):475-488.

Gallagher JE, Clarke W, Eaton KA, Wilson NH. 2009. A question of value: A qualitative study of vocational dental practitioners' views on oral healthcare systems and their future careers. Prim Dent Care. 16(1):29-37.

Gallagher JE, Wilson NH. 2009. The future dental workforce? Br Dent J. 206(4):195-199.

Gerritsen PF, Schrijvers AJ, Cune MS, van der Bilt A, de Putter C. 2014. Assessment of the oral health condition of nursing home residents by primary care nurses. Spec Care Dentist. 34(6):260-264.

Gerritsen PF, van der Bilt A, Cune MS, Schrijvers AJ, de Putter C. 2013. Integrated versus incidental dental care in nursing homes. Spec Care Dentist. 33(5):227-231.

Ghotane SG, Al-Haboubi M, Kendall N, Robertson C, Gallagher JE. 2015. Dentists with enhanced skills (special interest) in endodontics: Gatekeepers views in London. BMC Oral Health. 15(1):110.

Ghotane SG, Harrison V, Radcliffe E, Jones E, Gallagher JE. 2017. Enhanced skills in periodontology: Evaluation of a pilot scheme for general dental practitioners and dental care professionals in London. Br Dent J. 222(9):700-707.

Giles E, Gray-Burrows KA, Bhatti A, Rutter L, Purdy J, Zoltie T, Pavitt S, Marshman Z, West R, Day PF. 2021. "Strong teeth": An early-phase study to assess the feasibility of an oral health intervention delivered by dental teams to parents of young children. BMC Oral Health. 21(1):267.

Giles E, Rizvi Z, Gray JA, Barker CS, Spencer RJ. 2019. To accept, or not to accept? A service evaluation to appraise complexity assessment of orthodontic patients referred into a secondary care setting. Br Dent J. 226(12):963-966.

Gnich W, Sherriff A, Bonetti D, Conway DI, Macpherson LMD. 2018. The effect of introducing a financial incentive to promote application of fluoride varnish in dental practice in Scotland: A natural experiment. Implement Sci. 13(1):95.

Godson JH, Gallagher JE. 2021. Editorial - delivering better oral health 2021 - what's new and where next? Community Dent Health. 38(4):224-225.

Goldthorpe J, Sanders C, Gough L, Rogers J, Bridgman C, Tickle M, Pretty I. 2018. Implementing and evaluating a primary care service for oral surgery: A case study. BMC Health Serv Res. 18(1):636.

Goodwin N, Morris AJ, Hill KB, McLeod HS, Burke FJ, Hall AC. 2003. National evaluation of personal dental services (PDS) pilots: Main findings and policy implications. Br Dent J. 195(11):640-643.

Grønbæk AB, Petersen F, Haubek D, Poulsen S. 2017. Dentoalveolar oral surgery in children and adolescents: Organization and surgical treatment in a large, Danish municipal dental service. Acta Odontol Scand. 75(8):603-607.

Hall AC, Hill KB, Goodwin N, Morris AJ, Burke FJ. 2003. National evaluation of personal dental services: The perspective of dentists and professionals complementary to dentistry. Br Dent J. 195(11):651-653.

Harris R. 2003. Access to NHS dentistry in south cheshire: A follow up of people using telephone helplines to obtain NHS dental care. Br Dent J. 195(8):457-461; discussion 450.

Harris R, Brown S, Holt R, Perkins E. 2014. Do institutional logics predict interpretation of contract rules at the dental chair-side? Soc Sci Med. 122:81-89.

Harris R, Burnside G, Ashcroft A, Grieveson B. 2009. Job satisfaction of dental practitioners before and after a change in incentives and governance: A longitudinal study. Br Dent J. 207(2):E4; discussion 74-75.

Harris R, Lowers V, Laverty L, Vernazza C, Burnside G, Brown S, Ternent L. 2020. Comparing how patients value and respond to information on risk given in three different forms during dental check-ups: The prefer randomised controlled trial. Trials. 21(1):21.

Harris RV, Dancer JM, Montasem A. 2011. The impact of changes in incentives and governance on the motivation of dental practitioners. Int J Health Plann Manage. 26(1):70-88.

Harris RV, Dancer JM, Smith D, Campbell S. 2009. The use of conversation mapping to frame key perceptual issues facing the general dental practice system in England. Community Dent Health. 26(2):84-91.

Hearnshaw S. 2015. Local dental networks: Trendy or transformational? Community Dent Health. 32(4):194-195.

Hede B, Thiesen H, Christensen LB. 2019. A program review of a community-based oral health care program for socially vulnerable and underserved citizens in Denmark. Acta Odontol Scand. 77(5):364-370.

Hempel FM, Krois J, Paris S, Beuer F, Kuhlmey A, Schwendicke F. 2020. Prosthetic treatment patterns in the very old: An insurance database analysis from northeast Germany. Clin Oral Investig. 24(11):3981-3995.

Henschke C, Winkelmann J, Eriksen A, Orejas Pérez E, Klingenberger D. 2023. Oral health status and coverage of oral health care: A five-country comparison. Health Policy. 137:104913.

Hill H, Birch S, Tickle M, McDonald R, Donaldson M, O'Carolan D, Brocklehurst P. 2017. Does capitation affect the delivery of oral healthcare and access to services? Evidence from a pilot contact in Northern Ireland. BMC Health Serv Res. 17(1):175.

Hill H, Howarth E, Walsh T, Tickle M, Birch S, Brocklehurst P. 2020. The impact of changing provider remuneration on clinical activity and quality of care: Evaluation of a pilot NHS contract in Northern Ireland. Community Dent Oral Epidemiol. 48(5):395-401.

Hill H, Macey R, Brocklehurst P. 2017. A markov model assessing the impact on primary care practice revenues and patient's health when using mid-level providers, lesson learned from the United Kingdom. J Public Health Dent. 77(4):334-343.

Hill KB, Morris AJ, White DA, McHugh S, Atwal R, Burke FJ. 2009. A comparison of personal dental service (PDS) and general dental service (GDS) patients in terms of reported interventions, oral health and dentists' perceptions. Community Dent Health. 26(3):170-176.

Hill KB, Rimington D. 2011. Investigation of the oral health needs for homeless people in specialist units in London, Cardiff, Glasgow and Birmingham. Prim Health Care Res Dev. 12(2):135-144.

Hill KB, White DA, Morris AJ, Hall AC, Goodwin N, Burke FJ. 2003. National evaluation of personal dental services: A qualitative investigation into patients' perceptions of dental services. Br Dent J. 195(11):654-656.

Ho AYJ, Crawford F, Clarkson J. 2009. The use of the index of orthodontic treatment need in dental primary care. Br Dent J. 206(8):E16; discussion 418-419.

Hodge T. 2010. Orthodontic therapists--a challenge for the 21st century. J Orthod. 37(4):297-301.

Hodgins F, Sherriff A, Gnich W, Ross AJ, Macpherson LMD. 2018. The effectiveness of dental health support workers at linking families with primary care dental practices: A population-wide data linkage cohort study. BMC Oral Health. 18(1):191.

Holmes RD, Bate A, Steele JG, Donaldson C. 2009. Commissioning NHS dentistry in England: Issues for decision-makers managing the new contract with finite resources. Health Policy. 91(1):79-88.

Holmes RD, Donaldson C, Exley C, Steele JG. 2008. Managing resources in NHS dentistry: The views of decision-makers in primary care organisations. Br Dent J. 205(6):E11; discussion 328-329.

Holmes RD, Steele J, Exley CE, Donaldson C. 2011. Managing resources in NHS dentistry: Using health economics to inform commissioning decisions. BMC Health Serv Res. 11:138.

Holmes RD, Steele JG, Donaldson C, Exley C. 2015. Learning from contract change in primary care dentistry: A qualitative study of stakeholders in the north of England. Health Policy. 119(9):1218-1225.

Holm-Pedersen P, Vigild M, Nitschke I, Berkey DB. 2005. Dental care for aging populations in Denmark, Sweden, Norway, United Kingdom, and Germany. J Dent Educ. 69(9):987-997.

Howard-Williams P. 2009. Clinical audit and peer review scheme for the south west post-new 2006 dental contract: A report on progress so far. Br Dent J. 206(1):37-41.

Hulme C, Robinson PG, Saloniki EC, Vinall-Collier K, Baxter PD, Douglas G, Gibson B, Godson JH, Meads D, Pavitt SH. 2016. Shaping dental contract reform: A clinical and cost-effective analysis of incentive-driven commissioning for improved oral health in primary dental care. BMJ Open. 6(9):e013549.

Humphris GM, Zhou Y. 2014. Prediction of nursery school-aged children who refuse fluoride varnish administration in a community setting: A Childsmile investigation. Int J Paediatr Dent. 24(4):245-251.

Hussein RJ, Krohn R, Kaufmann-Kolle P, Willms G. 2017. Quality indicators for the use of systemic antibiotics in dentistry. Z Evid Fortbild Qual Gesundhwes. 122:1-8.

Ikenwilo D. 2013. A difference-in-differences analysis of the effect of free dental check-ups in Scotland. Soc Sci Med. 83:10-18.

Jackson RJ, Baird WO, Worthington LS, Robinson PG. 2007. A survey to investigate shortfalls in the dental care professional (DCP) workforce in south Yorkshire in 2004. Prim Dent Care. 14(4):129-135.

Jaggee G, Dooey J, Gallagher JE, Radford DR. 2019. Bouncing on the fringes of the dental system: Clinical dental technicians, a decade after their creation. Br Dent J. 226(6):432-436.

Jessop NM, Kay EJ, Mellor AC, Whittle JG, Jenner A. 2001. Management information failings and future requirements for dental commissioning groups. Br Dent J. 190(1):36-38.

John JH, Easterby-Smith V, Percival KR. 2014. Piloting a local dental network across Hampshire and isle of wight primary care trusts. Br Dent J. 217(5):E10.

Jones CM. 2001. Capitation registration and social deprivation in England. An inverse 'dental' care law? Br Dent J. 190(4):203-206.

Kalmus O, Chalkley M, Listl S. 2022. Effects of provider incentives on dental x-raying in NHS Scotland: What happens if patients switch providers? Eur J Health Econ. 23(1):59-65.

Kay E, Owen L, Taylor M, Claxton L, Sheppard L. 2018. The use of cost-utility analysis for the evaluation of caries prevention: An exploratory case study of two community-based public health interventions in a high-risk population in the UK. Community Dent Health. 35(1):30-36.

Kendall N. 2009. Improving access to oral surgery services in primary care. Prim Dent Care. 16(4):137-142.

Kidd JB, McMahon AD, Sherriff A, Gnich W, Mahmoud A, Macpherson LM, Conway DI. 2020. Evaluation of a national complex oral health improvement programme: A population data linkage cohort study in Scotland. BMJ Open. 10(11):e038116.

Kleinman ER, Harper PR, Gallagher JE. 2009. Trends in NHS primary dental care for older people in England: Implications for the future. Gerodontology. 26(3):193-201.

Lacey S. 2006. The relationship between NHS patient charges and the uptake of dental examinations by Scottish adults 1982-1998. Br Dent J. 201(6):361-364.

Lala R, Jones K. 2019. The use of the nice ten step model to conduct an oral health needs assessment in south Yorkshire and Bassetlaw. Community Dent Health. 36(1):5-8.

Lambert-Humble S. 2005. Opportunities for the dental team arising out of the new arrangements for primary dental care. Prim Dent Care. 12(1):15-19.

Landes DP. 2002. The provision of general anaesthesia in dental practice, an end which had to come? Br Dent J. 192(3):129-131.

Laverty L, Harris R. 2018. Can conditional health policies be justified? A policy analysis of the new NHS dental contract reforms. Soc Sci Med. 207:46-54.

Listl S, Chalkley M. 2014. Provider payment bares teeth: Dentist reimbursement and the use of check-up examinations. Soc Sci Med. 111:110-116.

Luciak-Donsberger C. 2003. Origins and benefits of dental hygiene practice in Europe. Int J Dent Hyg. 1(1):29-42.

Lynch M, Calnan M. 2003. The changing public/private mix in dentistry in the UK--a supply-side perspective. Health Econ. 12(4):309-321.

Macpherson LM, Anopa Y, Conway DI, McMahon AD. 2013. National supervised toothbrushing program and dental decay in Scotland. J Dent Res. 92(2):109-113.

Macpherson LM, Ball GE, Brewster L, Duane B, Hodges CL, Wright W, Gnich W, Rodgers J, McCall DR, Turner S, Conway DI. 2010. Childsmile: The national child oral health improvement programme in Scotland. Part 1: Establishment and development. Br Dent J. 209(2):73-78.

Mauthe PW, Eaton KA. 2011. An investigation into the bitewing radiographic prescribing patterns of west Kent general dental practitioners. Prim Dent Care. 18(3):107-114.

McDonald R, Cheraghi-Sohi S, Sanders C, Tickle M. 2012. Changes to financial incentives in English dentistry 2006-2009: A qualitative study. Community Dent Oral Epidemiol. 40(5):468-473.

McLeod HS, Morris AJ. 2003. Evaluation of personal dental services (PDS) first wave pilots: The alternative to general dental services (GDS) offered by the capitation-based pilots. Br Dent J. 195(11):644-650.

Mills I, Batchelor P. 2011. Quality indicators: The rationale behind their use in NHS dentistry. Br Dent J. 211(1):11-15.

Mills I, Frost J, Moles DR, Kay E. 2013. Patient-centred care in general dental practice: Sound sense or soundbite? Br Dent J. 215(2):81-85.

Milsom KM, Threlfall A, Pine K, Tickle M, Blinkhorn AS, Kearney-Mitchell P. 2008. The introduction of the new dental contract in England - a baseline qualitative assessment. Br Dent J. 204(2):59-62.

Murphy JM, Burch TE, Dickenson AJ, Wong J, Moore R. 2018. An evidence-based oral health promotion programme: Lessons from Leicester. Oral Dis. 24(1-2):38-43.

Murray JJ. 2003. NHS dentistry: Options for change. Impressions one year on. Br Dent J. 195(11):627-629.

Nash DA, Friedman JW, Kardos TB, Kardos RL, Schwarz E, Satur J, Berg DG, Nasruddin J, Mumghamba EG, Davenport ES, Nagel R. 2008. Dental therapists: A global perspective. Int Dent J. 58(2):61-70.

Ness AR, Wills AK, Waylen A, Al-Ghatam R, Jones TE, Preston R, Ireland AJ, Persson M, Smallridge J, Hall AJ et al. 2015. Centralization of cleft care in the UK. Part 6: A tale of two studies. Orthod Craniofac Res. 18 Suppl 2(Suppl 2):56-62.

Ness AR, Wills AR, Waylen A, Smallridge J, Hall AJ, Sell D, Sandy JR. 2018. Closing the loop on centralization of cleft care in the United Kingdom. Cleft Palate Craniofac J. 55(2):248-251.

Newton JT, Alexandrou B, Bate BD, Best H. 2006. A qualitative analysis of the planning, implementation and management of a PDS scheme: Lessons for local commissioning of dental services. Br Dent J. 200(11):625-630; discussion 618; quiz 638.

Nitschke I, Nitschke S, Haffner C, Sobotta BAJ, Jockusch J. 2022. On the necessity of a geriatric oral health care transition model: Towards an inclusive and resource-oriented transition process. Int J Environ Res Public Health. 19(10).

Nitschke I, Wendland A, Weber S, Jockusch J, Lethaus B, Hahnel S. 2021. Considerations for the prosthetic dental treatment of geriatric patients in Germany. J Clin Med. 10(2):304.

Northcott A, Brocklehurst P, Jerković-Ćosić K, Reinders JJ, McDermott I, Tickle M. 2013. Direct access: Lessons learnt from the Netherlands. Br Dent J. 215(12):607-610.

Olajide OJ, Shucksmith J, Maguire A, Zohoori FV. 2017. Using normalisation process theory to investigate the implementation of school-based oral health promotion. Community Dent Health. 34(3):137-142.

O'Malley L, Worthington HV, Donaldson M, O'Neil C, Birch S, Noble S, Killough S, Murphy L, Greer M, Brodison J et al. 2018. Oral health behaviours of parents and young children in a practice-based caries prevention trial in Northern Ireland. Community Dent Oral Epidemiol. 46(3):251-257.

Ormond C, Douglas G, Pitts N. 2010. The use of the international caries detection and assessment system (ICDAS) in a national health service general dental practice as part of an oral health assessment. Prim Dent Care. 17(4):153-159.

Overs E, Woods C, Jones L, Williams L, Williams S, Burton C, Brocklehurst PR. 2023. Using arts-based research in applied health care: An example from an evaluation of NHS dental contract reform in Wales. J Health Serv Res Policy. 28(3):190-196.

Owen C, Seddon C, Clarke K, Bysouth T. 2019. NHS general dentistry in Wales: Evaluation of patient access and budget expenditure. Br Dent J. 226(12):967-978.

Page J, Weld JA, Kidd EA. 2010. Caries control in health service practice. Br Dent J. 208(10):449-450.

Paige CJ, Shahid SK. 2014. Developing and implementing a fluoride varnish programme for young children in Bradford, UK. Community Dent Health. 31(1):5-8.

Patel A, McQuillan J, Johnson J, Sadio H, Dungarwalla M. 2023. Project tooth fairy: A pan-London initiative from conception to delivery to patient-reported experience measures. Br Dent J. 234(10):739-745.

Patel PM, Lynch CD, Sloan AJ, Gilmour AS. 2010. Treatment planning for replacing missing teeth in UK general dental practice: Current trends. J Oral Rehabil. 37(7):509-517.

Patel RN, Antonarakis GS. 2013. Factors influencing the adoption and implementation of teledentistry in the UK, with a focus on orthodontics. Community Dent Oral Epidemiol. 41(5):424-431.

Pau A, Nanjappa S, Diu S. 2010. Evaluation of dental practitioners with special interest in minor oral surgery. Br Dent J. 208(3):103-107.

Pavitt SH, Baxter PD, Brunton PA, Douglas G, Edlin R, Gibson BJ, Godson J, Hall M, Porritt J, Robinson PG et al. 2014. The incentive protocol: An evaluation of the organisation and delivery of NHS dental healthcare to patients-innovation in the commissioning of primary dental care service delivery and organisation in the UK. BMJ Open. 4(9):e005931.

Perry JG. 2011. A preliminary investigation into the effect of the use of the short message service (SMS) on patient attendance at an NHS dental access centre in Scotland. Prim Dent Care. 18(4):145-149.

Persson M, Sandy JR, Waylen A, Wills AK, Al-Ghatam R, Ireland AJ, Hall AJ, Hollingworth W, Jones T, Peters TJ et al. 2015. A cross-sectional survey of 5-year-old children with non-syndromic unilateral cleft lip and palate: The cleft care UK study. Part 1: Background and methodology. Orthod Craniofac Res. 18 Suppl 2(Suppl 2):1-13.

Petersen PE, Davidsen M, Jensen HAR, Ekholm O, Christensen AI. 2021. Trends in dentate status and preventive dental visits of the adult population in Denmark over 30 years (1987–2017). Eur J Oral Sci. 129(5).

Petersen PE, Kjøller M, Christensen LB, Krustrup U. 2004. Changing dentate status of adults, use of dental health services, and achievement of national dental health goals in Denmark by the year 2000. J Public Health Dent. 64(3):127-135.

Pitts NB. 2003. NHS dentistry: Options for change in context--a personal overview of a landmark document and what it could mean for the future of dental services. Br Dent J. 195(11):631-635.

Richards W, Filipponi T, Coll AM. 2020. General dental practice and improved oral health: Is there a win-win for both the professional establishment and government? Br Dent J. 228(8):581-585.

Richards W, Gear T. 2008. Changes in the balance between dentists, patients and funders in the NHS and their consequences. Prim Dent Care. 15(1):13-16.

Richmond S, Karki A. 2012. Complexities associated with orthodontic services in the national health service. Br Dent J. 212(3):E5.

Robinson PG, Douglas GVA, Gibson BJ, Godson J, Vinall-Collier K, Pavitt S, Hulme C. 2019. Remuneration of primary dental care in England: A qualitative framework analysis of perspectives of a new service delivery model incorporating incentives for improved access, quality and health outcomes. BMJ Open. 9(10):e031886.

Rodgers J, Macpherson LM. 2006. General dental practitioners' perceptions of the west of Scotland cancer awareness programme oral cancer campaign. Br Dent J. 200(12):693-697; discussion 675.

Rodgers J, Macpherson LM, Smith GL, Crighton AJ, Carton AT, Conway DI. 2007. Characteristics of patients attending rapid access clinics during the west of Scotland cancer awareness programme oral cancer campaign. Br Dent J. 202(11):E28; discussion 680-681.

Rosing K, Leggett H, Csikar J, Vinall-Collier K, Christensen LB, Whelton H, Douglas GVA. 2019. Barriers and facilitators for prevention in Danish dental care. Acta Odontol Scand. 77(6):439-451.

Ross AJ, Sherriff A, Kidd J, Deas L, Eaves J, Blokland A, Wright B, King P, McMahon AD, Conway DI, Macpherson LMD. 2023. Evaluating Childsmile, Scotland’s national oral health improvement programme for children. Community Dent Oral Epidemiol. 51(1):133-138.

Ross M, Turner S. 2015. Direct access in the UK: What do dentists really think? Br Dent J. 218(11):641-647.

Ross MK, Turner S, Ibbetson RJ. 2012. The impact of general dental council registration and continuing professional development on UK dental care professionals: (2) dental technicians. Br Dent J. 213(8):E13.

Saekel R. 2010. China's oral care system in transition: Lessons to be learned from Germany. Int J Oral Sci. 2(3):158-176.

Sagheri D, Hahn P, Hellwig E. 2007. Assessing the oral health of school-age children and the current school-based dental screening programme in Freiburg (Germany). Int J Dent Hyg. 5(4):236-241.

Salomon-Ibarra CC, Ravaghi V, Hill K, Jones CM, Landes DP, Morris AJ. 2019. Low rates of dental attendance by the age of one and inequality between local government administrative areas in England. Community Dent Health. 36(1):22-26.

Sandy J, Rumsey N, Persson M, Waylen A, Kilpatrick N, Ireland T, Ness A. 2012. Using service rationalisation to build a research network: Lessons from the centralisation of UK services for children with cleft lip and palate. Br Dent J. 212(11):553-555.

Schorer-Jensma MA, Veerkamp JS. 2010. A comparison of paediatric dentists' and general dental practitioners' care patterns in paediatric dental care. Eur Arch Paediatr Dent. 11(2):93-96.

Schuller AA, van Dommelen P, Poorterman JH. 2014. Trends in oral health in young people in the Netherlands over the past 20 years: A study in a changing context. Community Dent Oral Epidemiol. 42(2):178-184.

Schwendicke F, Göstemeyer G, Stolpe M, Krois J. 2018. Amalgam alternatives: Cost-effectiveness and value of information analysis. J Dent Res. 97(12):1317-1323.

Searle A, Scott JK, Sandy J, Ness A, Waylen A. 2015. Clinical directors' views of centralisation and commissioning of cleft services in the UK. BMC Oral Health. 15:12.

Sell D, Mildinhall S, Albery L, Wills AK, Sandy JR, Ness AR. 2015. The cleft care UK study. Part 4: Perceptual speech outcomes. Orthod Craniofac Res. 18 Suppl 2(Suppl 2):36-46.

Seward M. 2000. Dental education in the 21st century. Prim Dent Care. 7(1):5-7.

Simons D, Pearson N, Evans P, Wallace T, Eke M, Wright D. 2015. Improving access to dental care for vulnerable children; further development of the back2school programme in 2013. Community Dent Health. 32(2):68-71.

Simons D, Pearson N, Movasaghi Z. 2012. Developing dental services for homeless people in east London. Br Dent J. 213(7):E11.

Sinclair E, Eaton KA, Widström E. 2019. The healthcare systems and provision of oral healthcare in European Union member states. Part 10: Comparison of systems and with the United Kingdom. Br Dent J. 227(4):305-310.

Skeie MS, Klock KS. 2014. Scandinavian systems monitoring the oral health in children and adolescents; an evaluation of their quality and utility in the light of modern perspectives of caries management. BMC Oral Health. 14:43.

Slator R, Perisanidou LI, Sell D, Sandy J, Ness AR, Wills AK. 2023. Surgical sequence, timing and volume, and variation in dento-facial outcome, speech and secondary surgery in children with unilateral cleft lip and palate: The cleft care UK study. Orthod Craniofac Res. 26(2):297-309.

Slator R, Perisanidou LI, Waylen A, Sandy J, Ness A, Wills AK. 2020. Range and timing of surgery, and surgical sequences used, in primary repair of complete unilateral cleft lip and palate: The cleft care UK study. Orthod Craniofac Res. 23(2):166-173.

Smallridge J, Hall AJ, Chorbachi R, Parfect V, Persson M, Ireland AJ, Wills AK, Ness AR, Sandy JR. 2015. Functional outcomes in the cleft care UK study--part 3: Oral health and audiology. Orthod Craniofac Res. 18 Suppl 2(Suppl Suppl 2):25-35.

Smits KPJ, Listl S, Plachokova AS, Van der Galien O, Kalmus O. 2020. Effect of periodontal treatment on diabetes-related healthcare costs: A retrospective study. BMJ Open Diabetes Res Care. 8(1).

Spinler K, Aarabi G, Valdez R, Kofahl C, Heydecke G, König HH, Hajek A. 2019. Prevalence and determinants of dental visits among older adults: Findings of a nationally representative longitudinal study. BMC Health Serv Res. 19(1):590.

Stewart M, Keightley A, Maguire A, Chadwick B, Vale L, Homer T, Douglas G, Deery C, Marshman Z, Ryan V, Innes N. 2015. Investigating the management of carious primary teeth in general dental practice: An overview of the development and conduct of the fiction trial. Prim Dent J. 4(4):67-73.

Strippel H. 2010. Effectiveness of structured comprehensive paediatric oral health education for parents of children less than two years of age in Germany. Community Dent Health. 27(2):74-80.

Sturrock A, Preshaw PM, Hayes C, Wilkes S. 2020. 'We do not seem to engage with dentists': A qualitative study of primary healthcare staff and patients in the north east of England on the role of pharmacists in oral healthcare. BMJ Open. 10(2):e032261.

Sun N, Burnside G, Harris R. 2010. Patient satisfaction with care by dental therapists. Br Dent J. 208(5):E9; discussion 212-213.

Sun N, Harris RV. 2011. Models of practice organisation using dental therapists: English case studies. Br Dent J. 211(3):E6.

Sundby A, Petersen PE. 2003. Oral health status in relation to ethnicity of children in the municipality of Copenhagen, Denmark. Int J Paediatr Dent. 13(3):150-157.

Sweeney MP, Williams C, Kennedy C, Macpherson LM, Turner S, Bagg J. 2007. Oral health care and status of elderly care home residents in Glasgow. Community Dent Health. 24(1):37-42.

Telford C, Murray L, Donaldson M, O'Neill C. 2012. An analysis examining socio-economic variations in the provision of NHS general dental practitioner care under a fee for service contract among adolescents: Northern Ireland longitudinal study. Community Dent Oral Epidemiol. 40(1):70-79.

Templeton AR, Young L, Bish A, Gnich W, Cassie H, Treweek S, Bonetti D, Stirling D, Macpherson L, McCann S et al. 2016. Patient-, organization-, and system-level barriers and facilitators to preventive oral health care: A convergent mixed-methods study in primary dental care. Implement Sci. 11:5.

Thornley PH, Stewardson DA, Rout PG, Burke FJ. 2004. Rectangular collimation and radiographic efficacy in eight general dental practices in the West Midlands. Prim Dent Care. 11(3):81-86.

Tickle M. 2012. Revolution in the provision of dental services in the UK. Community Dent Oral Epidemiol. 40 Suppl 2:110-116.

Tickle M, McDonald R, Franklin J, Aggarwal VR, Milsom K, Reeves D. 2011. Paying for the wrong kind of performance? Financial incentives and behaviour changes in national health service dentistry 1992-2009. Community Dent Oral Epidemiol. 39(5):465-473.

Tickle M, Milsom KM, Buchanan K, Blinkhorn AS. 2006. Dental screening in schools: The views of parents, teachers and school nurses. Br Dent J. 201(12):769-773; discussion 767.

Tickle M, Milsom KM, Donaldson M, Killough S, O'Neill C, Crealey G, Sutton M, Noble S, Greer M, Worthington HV. 2011. Protocol for Northern Ireland caries prevention in practice trial (NIC-PIP) trial: A randomised controlled trial to measure the effects and costs of a dental caries prevention regime for young children attending primary care dental services. BMC Oral Health. 11:27.

Topping GV. 2005. Out-of-hours emergency dental services--evaluation of the first year of a pilot project in fife. Br Dent J. 198(4):193-197.

Trescher AL, Listl S, van der Galien O, Gabel F, Kalmus O. 2020. Once bitten, twice shy? Lessons learned from an experiment to liberalize price regulations for dental care. Eur J Health Econ. 21(3):425-436.

Tulip DE, Palmer NO. 2008. A retrospective investigation of the clinical management of patients attending an out of hours dental clinic in merseyside under the new NHS dental contract. Br Dent J. 205(12):659-664; discussion 648.

Turner S, Brewster L, Kidd J, Gnich W, Ball GE, Milburn K, Pitts NB, Goold S, Conway DI, Macpherson LM. 2010. Childsmile: The national child oral health improvement programme in Scotland. Part 2: Monitoring and delivery. Br Dent J. 209(2):79-83.

Turner S, Ross MK, Ibbetson RJ. 2012. The impact of general dental council registration and continuing professional development on UK dental care professionals: (1) dental nurses. Br Dent J. 213(2):E2.

van der Poel C. 2006. Research defines public dental health promotion in youth. Int J Dent Hyg. 4(1):24-29.

Verlinden DA, Reijneveld SA, Lanting CI, van Wouwe JP, Schuller AA. 2019. Socio-economic inequality in oral health in childhood to young adulthood, despite full dental coverage. Eur J Oral Sci. 127(3):248-253.

Vernazza CR, Carr K, Wildman J, Gray J, Holmes RD, Exley C, Smith RA, Donaldson C. 2018. Resource allocation in NHS dentistry: Recognition of societal preferences (raindrop): Study protocol. BMC Health Serv Res. 18(1):487.

Vernazza CR, Rousseau N, Steele JG, Ellis JS, Thomason JM, Eastham J, Exley C. 2015. Introducing high-cost health care to patients: Dentists' accounts of offering dental implant treatment. Community Dent Oral Epidemiol. 43(1):75-85.

Vernazza CR, Taylor G, Donaldson C, Gray J, Holmes R, Carr K, Exley C. 2019. How does priority setting for resource allocation happen in commissioning dental services in a nationally led, regionally delivered system: A qualitative study using semistructured interviews with NHS England dental commissioners. BMJ Open. 9(3):e024995.

Wagner Y, Heinrich-Weltzien R. 2016. Evaluation of an interdisciplinary preventive programme for early childhood caries: Findings of a regional German birth cohort study. Clin Oral Investig. 20(8):1943-1952.

Wagner Y, Heinrich-Weltzien R. 2017. Evaluation of a regional German interdisciplinary oral health programme for children from birth to 5 years of age. Clin Oral Investig. 21(1):225-235.

Wagner Y, Knaup I, Knaup TJ, Jacobs C, Wolf M. 2020. Influence of a programme for prevention of early childhood caries on early orthodontic treatment needs. Clin Oral Investig. 24(12):4313-4324.

Wang NJ, Petersen PE, Sveinsdóttir EG, Arnadóttir IB, Källestål C. 2018. Recall intervals and time used for examination and prevention by dentists in child dental care in Denmark, Iceland, Norway and Sweden in 1996 and 2014. Community Dent Health. 35(1):52-57.

Wanyonyi KL, Radford DR, Gallagher JE. 2013. The relationship between access to and use of dental services following expansion of a primary care service to embrace dental team training. Public Health. 127(11):1028-1033.

Wanyonyi KL, Radford DR, Harper PR, Gallagher JE. 2015. Alternative scenarios: Harnessing mid-level providers and evidence-based practice in primary dental care in England through operational research. Hum Resour Health. 13:78.

Watson M. 2003. The health and social care bill. Br Dent J. 195(11):637-638.

Watt RG, Stillman-Lowe C, Munday P, Plimley W, Fuller SS. 2001. The development of a national oral health promotion programme for pre-school children in England. Int Dent J. 51(5):334-338.

Weening-Verbree LF, Schuller DAA, Cheung SL, Zuidema P, Schans P, Hobbelen D. 2021. Barriers and facilitators of oral health care experienced by nursing home staff. Geriatr Nurs. 42(4):799-805.

Welsh S, Edwards M, Hunter L. 2012. Caring for smiles--a new educational resource for oral health training in care homes. Gerodontology. 29(2):e1161-1162.

Wessels M, Knappe D. 2008. Effects of the new fixed-subsidy system for prosthetic dental care in Germany: Results of descriptive research. Int Dent J. 58(1):29-35.

Whittaker W, Birch S. 2012. Provider incentives and access to dental care: Evaluating NHS reforms in England. Soc Sci Med. 75(12):2515-2521.

Whittle JG. 2000. The provision of primary care dental general anaesthesia and sedation in the north west region of England, 1996-1999. Br Dent J. 189(9):500-502.

Whittle JG, Haworth JL. 2000. Maintaining good dental practice: The east Lancashire approach to dentists whose performance gives cause for concern. Br Dent J. 188(10):539-542.

Willett JA, Palmer NO. 2009. An investigation of the attitudes and fears of vocational dental practitioners in England and Wales in 2007. Prim Dent Care. 16(3):103-110.

Winkelmann J, Gómez Rossi J, Schwendicke F, Dimova A, Atanasova E, Habicht T, Kasekamp K, Gandré C, Or Z, McAuliffe Ú et al. 2022. Exploring variation of coverage and access to dental care for adults in 11 European countries: A vignette approach. BMC Oral Health. 22(1):65.

Winkelmann, Rossi, Van Ginneken. 2022. Oral health care in Europe: financing, access and provision. World Health Organization Report No.: 35833482. [accessed 2023 Jan 5]. https://apps.who.int/iris/bitstream/handle/10665/355605/HiT-24-2-2022-eng.pdf?sequence=1&isAllowed=y.

Winter J, Bartsch B, Schütz C, Jablonski-Momeni A, Pieper K. 2019. Implementation and evaluation of an interdisciplinary preventive program to prevent early childhood caries. Clin Oral Investig. 23(1):187-197.

Wright D, Batchelor PA. 2002. General dental practitioners' beliefs on the perceived effects of and their preferences for remuneration mechanisms. Br Dent J. 192(1):46-49.

Ziller S, Eaton KE, Widström E. 2015. The healthcare system and the provision of oral healthcare in European Union member states. Part 1: Germany. Br Dent J. 218(4):239-244.
